# Psychosocial and Socio-Economic Crisis in Bangladesh Due to COVID-19 Pandemic: A Perception-Based Assessment

**DOI:** 10.3389/fpubh.2020.00341

**Published:** 2020-06-26

**Authors:** Md. Bodrud-Doza, Mashura Shammi, Laura Bahlman, Abu Reza Md. Towfiqul Islam, Md. Mostafizur Rahman

**Affiliations:** ^1^Climate Change Programme, BRAC, Dhaka, Bangladesh; ^2^Department of Environmental Sciences, Jahangirnagar University, Dhaka, Bangladesh; ^3^International Centre for Climate Change and Development (ICCCAD), Independent University Bangladesh (IUB), Dhaka, Bangladesh; ^4^Department of Disaster Management, Begum Rokeya University, Rangpur, Bangladesh

**Keywords:** COVID-19, perception-based questionnaire, principal component analysis (PCA), linear regression model, fear, social conflict

## Abstract

**Background:** The spread of the COVID-19 pandemic, the partial lockdown, the disease intensity, weak governance in the healthcare system, insufficient medical facilities, unawareness, and the sharing of misinformation in the mass media has led to people experiencing fear and anxiety. The present study intended to conduct a perception-based analysis to get an idea of people's psychosocial and socio-economic crisis, and the possible environmental crisis, amidst the COVID-19 pandemic in Bangladesh.

**Methods:** A perception-based questionnaire was put online for Bangladeshi citizens of 18 years and/or older. The sample size was 1,066 respondents. Datasets were analyzed through a set of statistical techniques including principal component and hierarchical cluster analysis.

**Results:** There was a positive significant association between fear of the COVID-19 outbreak with the struggling healthcare system (*p* < 0.05) of the country. Also, there was a negative association between the fragile health system of Bangladesh and the government's ability to deal with the pandemic (*p* < 0.05), revealing the poor governance in the healthcare system. A positive association of shutdown and social distancing with the fear of losing one's own or a family members' life, influenced by a lack of healthcare treatment (*p* < 0.05), reveals that, due to the decision of shutting down normal activities, people may be experiencing mental and economic stress. However, a positive association of the socio-economic impact of the shutdown with poor people's suffering, the price hike of basic essentials, the hindering of formal education (*p* < 0.05), and the possibility of a severe socio-economic and health crisis will be aggravated. Moreover, there is a possibility of a climate change-induced disaster and infectious diseases like dengue during/after the COVID-19 situation, which will create severe food insecurity (*p* < 0.01) and a further healthcare crisis.

**Conclusions:** The partial lockdown in Bangladesh due to the COVID-19 pandemic increased community transmission and worsened the healthcare crisis, economic burden, and loss of GDP despite the resuming of industrial operations. In society, it has created psychosocial and socio-economic insecurity among people due to the loss of lives and livelihoods. The government should take proper inclusive steps for risk assessment, communications, and financial stimulus toward the public to alleviate their fear and anxiety, and to take proper action to boost mental health and well-being.

## Introduction

The novel coronavirus disease (COVID-19) began spreading in November 2019, in Wuhan, China. Following this, the World Health Organization (WHO) announced COVID-19 as a global pandemic on March 11th, 2020 (1). COVID-19 has advanced into a pandemic, starting initially as small clusters of transmission that combined into larger clusters in many countries, subsequently resulting in a widespread transmission (2). Social isolation, institutional and home quarantine, social distancing, and community containment measures were applied without delay (3). Through quick administrative action and raising awareness for individuals on social-distancing, stringent steps were taken to manage the spread of the disease by canceling thousands of locations that involved social gathering including offices, classrooms, reception centers, clubs, transport services, and travel restrictions, leaving many countries in complete lockdown (4). The remarkable actions and ventures in public health to quarantine mass numbers has prevented this virus from spreading exponentially between humans in China, Singapore, Hong Kong, and South Korea, despite initial cases (2, 5).

However, a surge of COVID-19 outbreaks in all inhabitable continents, with 84,187 deaths alone in the USA, indicates that the infection had passed the tipping point (1, 6). Today, as of the 26th of May 2020, total global COVID-19 cases have risen to 5,637,381, with the total number of deaths escalating to 3,49,291 (7). The accelerating spread of COVID-19 and its outcomes around the world has led people to experiencing fear, panic, concern, anxiety, stigma, depression, racism, and xenophobia (8). Bangladesh confirmed their first COVID-19 case on the 8th of March 2020 (9), followed by a nationwide lockdown from 26 March which had been extended several times until 30th May 2020 to prevent human transmission. The government deployed armed forces to facilitate social distancing on March 24th. Emergency healthcare services and law enforcement were exempt from this announcement. Yet more than 11 million people left Dhaka to return to their home districts and thus helped spread the diseases nationwide. Moreover, from the 25th of April 2020, all ready-made-garment (RMG) factories, industries, private offices, and business centers were allowed to open, leading to a “partial lockdown” in the country. The migration of RMG workers to the industrial districts and less community awareness about the disease has increased the transmission among millions of people.

**Figure 1:**
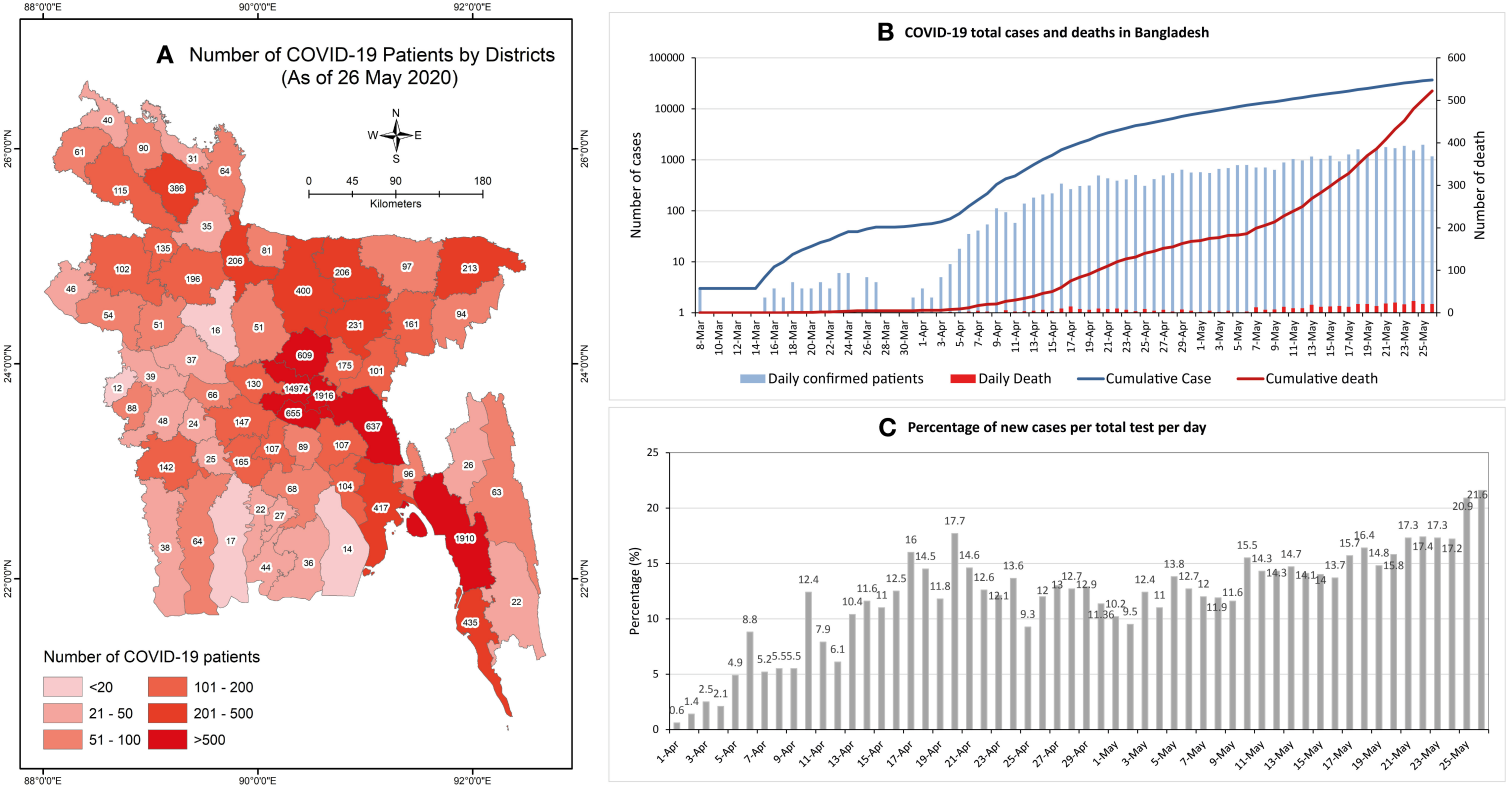
**(A)** Map of the study area showing the number of COVID-19 confirmed patients in the 64 districts of Bangladesh. **(B)** Daily confirmed COVID-19 patients and death count. **(C)** Percentage of new cases per total test per day (Data source: IEDCR).

The Institute of Epidemiology Disease Control and Research (IEDCR), under the Ministry of Health and Family Welfare (MFHW) and Directorate General of Health Services (DGHS), is responsible for researching epidemiological and communicable diseases such as COVID-19 in Bangladesh, as well as disease control and surveillance. Initially, IEDCR was the single and centralized laboratory for COVID-19 testing in Bangladesh (9). The DGHS, on the other hand, is the responsible body for the coordination of testing and sample collections of COVID-19 patients (10). As of the 26th of May 2020, according to IEDCR, the total number of COVID-19 positive cases stands at 36,751 with 522 deaths (Figures 1A,B). According to IEDCR, those aged between 21 and 40 are with the highest number of cases (55%), while those aged above 60 have had fatal cases of the disease (42%). At present, the fatality rate in Bangladesh is 1.41% (26th May 2020) which was initially 10.4% (8th April 2020) (9).

Although the number of laboratories for COVID-19 testing has increased to 48, all these labs are in major urban areas of Bangladesh and to get tested requires long waiting hours. More often the tests have been done after the patients had died. Very recently, more than 15% of those tested daily have tested as positive (Figure 1C), and the ratio of testing is 1,620/1 million people. In addition, it also takes a long time to get the result of the tests. Furthermore, there are only 1,169 Intensive Care Unit (ICU) beds in the country, of which 432 beds are in government hospitals and 737 in private hospitals. It is predicted that as the number of patients rise, the required number of ICU facilities will not be adequate (9). In addition, the healthcare staff and doctors were given low quality/no personal protective equipment (PPE) which has caused a high infection rate among them (11). Moreover, as laboratory staff, healthcare staff, and doctors have become increasingly infected, there is also a shortage of specialized trained personal to perform COVID-19 tests, meaning patient treatment will be disrupted.

Amidst the lockdown, due to the fear of contact transmission, private hospitals and clinics are not providing any services (11). The shortage of healthcare facilities for primary and critical care patients have therefore been depleted. The healthcare workers who have treated patients and become infected have been criticized socially and have faced social stigma from local people. In many locations public protests were observed against the establishment of quarantine facilities, COVID-19 care hospitals, and clinics. Social humiliation was a common practice of law enforcement authorities and government officials. On many occasions, family members left the infected and the deceased in the hospitals. The deceased were even denied burials in local graveyards, which are basic cultural rights as a Muslim (12). Moreover, the lockdown hit hard for those who earn daily wages and low and middle-income people who lost their jobs and their income source. The anxiety and fear of death from hunger or death from infection led to several suicide cases (13). Predictably, any contagious epidemic outbreak has harmful effects on individuals and society (14). Considering the population density, educational status, social structure, cultural norms, healthcare capacity, and often flawed policies taken by the Government of Bangladesh, it is hard to lock down a country of 165 million people. Moreover, Bangladesh hosts the largest refugee camps in the world in the Cox's Bazar district. The Rohingya refugees who fled from Myanmar reside in the camps of Cox's Bazar. 21 confirmed cases were found in the camps while the district had reported 435 confirmed cases (9). This depicts the scenario of public anxiety which should be immediately dealt with by the Government, along with the alliance groups, with proper information.

Amidst the current societal levels of anxiety and fear, the possibility of natural disasters such as tropical cyclones and monsoon floods and the potential for a dengue outbreak, seasonal influenza, or other infections are potentially overlooked. Furthermore, the consequences of incorrect disposal of used personal protective equipment (PPE) from COVID-19 hospitals without proper treatment in landfill sites has the potential for further disease transmission among the waste management personal and further environmental transmission. Considering the given circumstance, this study was designed to analyze the psychosocial, socio-economic, and possible environmental crisis based on public perception in Bangladesh due to the COVID-19 outbreak. This assessment may inform the government and policymakers of countries with a similar socioeconomic and cultural structure to Bangladesh.

## Methodology

### Study Procedure

To understand the possible psychosocial, socio-economic, and environmental impact of the COVID-19 outbreak in Bangladesh, we considered and identified several relevant and possible items based on the socio-economic situation, political analysis, the existing healthcare system, environmental analysis, possible emerging issues utilized from scenario developments, analysis of local and global reports of the COVID-19 pandemic from the print and electronic media, and a literature review. We prepared the questionnaire considering the demographic characteristics of Bangladesh, societal mental health conditions (MH), the healthcare system in Bangladesh (HSB), governance and political issues (GPI), socio-economic issues (SEI), immediate emerging issues (IEI) and enduring emerging issues (EEI). A total of 46 items were considered in the drafted questionnaires to understand people's perception of the COVID-19 outbreak in Bangladesh. Furthermore, expert consultation was considered to set and validate these 46 items.

We prepared the online-based questionnaire through Google to operate the survey nationwide. An introductory paragraph describing the objective of the questionnaire was shared with the respondents through email and through social platforms commonly used by Bangladeshi groups on Facebook, Messenger, LinkedIn, and WhatsApp. Relevant people were selected for targeted sampling. An online database of target participants was prepared by reviewing relevant websites and online social platforms of different groups in Bangladesh. The sample group was targeted considering Bangladeshi citizenship, their age, current activities, occupation, social and economic responsibilities, and engagement related to COVID-19 response, planning, and policymaking. The questionnaire survey was conducted from 28 March to 30 March 2020 during the lockdown period. The respondents belonged to different social categories, such as university faculty members and scholars, government officials, development workers or practitioners, doctors, engineers and technologists, youth leaders and students, businessmen and industry officials, banking and finance corporates, and independent researchers, among others. The answers to the survey questionnaire were voluntary. Data from 1082 respondents were collected through this online survey initially using the simple random sampling method following Keeble et al. (15). Following the removal of 16 incomplete questionnaires, 1,066 responses were finally retained for this study. A five-point (1 to 5) Likert scale was used for testing the statement descriptions that ranged from strongly disagree to strongly agree with the statements (Table 1).

**Table 1:** Descriptive statistics and item-total correlation.

**Sector**	**Statement**	**Denote**	**Strongly disagree % (*n*)**	**Disagree % (*n*)**	**Neither agree nor disagree % (*n*)**	**Agree % *(n)***	**Strongly agree% *(n)***	**Mean**	**Std. deviation**	**Skewness**	**Kurtosis**	**Corrected item-total correlation**
Mental health condition (MH)	I am afraid of the recent outbreak of coronavirus in Bangladesh	MH1	2.9 (310)	5 (53)	12.4 (132)	33.5 (357)	46.2 (493)	4.152	1.012	−1.271	1.203	0.381
	I am afraid of getting infected with coronavirus	MH2	3.7 (39)	7.8 (83)	22.3 (238)	33.5 (357)	32.7 (349)	3.839	1.080	−0.747	−0.07	0.355
	I am afraid of losing my life or my relatives' lives due to this outbreak	MH3	3.4 (36)	7 (75)	13.8 (147)	29.3 (312)	46.5 (496)	4.085	1.087	−1.131	0.525	0.360
	All the news of infection and deaths from COVID-19 in different media is increasing my fear	MH4	3.2 (34)	5.9 (63)	15 (160)	32.4 (345)	43.5 (464)	4.071	1.051	−1.11	0.659	0.376
	It makes me uncomfortable to be detached from regular activities due to lockdown	MH5	3.6 (38)	4.6 (49)	12.3 (131)	27.9 (297)	51.7 (551)	4.195	1.052	−1.369	1.301	0.344
Healthcare system in Bangladesh (HSB)	The healthcare system of Bangladesh is too fragile to deal with the recent outbreak of COVID-19	HSB1	3.2 (34)	3.8 (41)	8.7 (93)	22.3 (238)	61.9 (660)	4.359	1.010	−1.736	2.476	0.360
	A huge population is a pressure to the existing healthcare system to deal with COVID-19	HSB2	2.3 (25)	2.1 (22)	5.3 (57)	22.2 (237)	68 (725)	4.515	0.873	−2.237	5.195	0.421
	There is a lack of awareness of basic healthcare issues amongst most of the citizens of Bangladesh	HSB3	1.3 (14)	1.3 (14)	5.9 (63)	24.1 (257)	67.4 (718)	4.549	0.776	−2.147	5.378	0.456
	There is a lack of trained doctors and healthcare professionals to deal with the COVID-19	HSB4	2.9 (31)	3.8 (41)	13 (139)	28 (299)	52.2 (556)	4.227	1.008	−1.374	1.451	0.305
	There is a lack of healthcare facilities needed to combat the COVID-19 outbreak in Bangladesh	HSB5	1.3 (14)	0.9 (10)	4.8 (51)	17.5 (187)	75.4 (804)	4.648	0.734	−2.645	8.058	0.537
	There is a lack of healthcare infrastructure to deal with COVID-19	HSB6	1.3 (14)	1.8 (19)	4.9 (52)	19 (203)	73 (778)	4.606	0.776	−2.432	6.482	0.511
	There is a severe lack of bio-medical and hospital waste management facilities in Bangladesh	HSB7	0.7 (7)	1.1 (12)	7.3 (78)	22.4 (239)	68.5 (730)	4.569	0.734	−1.927	4.103	0.533
	There is a lack of COVID-19 testing facilities in Bangladesh	HSB8	1.6 (17)	0.9 (10)	3.1 (33)	12.4 (132)	82 (874)	4.722	0.715	−3.295	12.001	0.508
	There is a lack of budget or financial support in response to this outbreak	HSB9	5.3 (57)	7.9 (84)	11.9 (127)	25.7 (274)	49.2 (524)	4.054	1.186	−1.162	0.354	0.309
	Most of the poor people will not have access to existing healthcare facilities if they are infected by COVID-19	HSB10	1.1 (12)	1 (11)	6.1 (65)	15.9 (170)	75.8 (808)	4.643	0.741	−2.495	6.901	0.554
Governance and Political issues (GPI)	The Bangladesh government can deal with this outbreak	GPI1	28 (299)	26.2 (279)	21.9 (233)	15 (160)	8.9 (95)	2.506	1.284	0.436	−0.905	−0.054
	The government is taking this outbreak seriously	GPI2	19.9 (212)	25.2 (269)	22.5 (240)	23.5 (251)	8.8 (94)	2.762	1.257	0.121	−1.088	−0.011
	The government is making proper decisions in the right time	GPI3	32.6 (347)	30.7 (327)	19.1 (204)	12.3 (131)	5.3 (57)	2.272	1.190	0.664	−0.52	−0.078
	The government is involving other sectoral actors to combat the COVID-19 outbreak	GPI4	12.3 (131)	18.9 (202)	36.1 (385)	23.8 (254)	8.8 (94)	2.979	1.128	−0.105	−0.654	0.040
	The government needs support from the people to reduce the impact of COVID-19	GPI5	1.6 (17)	1 (11)	5.2 (55)	23.6 (252)	68.6 (731)	4.566	0.776	−2.319	6.367	0.435
	The government needs to formulate a policy and action plan and implement it immediately	GPI6	1 (11)	0.8 (9)	4.1 (44)	17.5 (187)	76.5 (815)	4.675	0.690	−2.73	8.888	0.499
	Developed nations are going to support Bangladesh in response to COVID-19	GPI7	4.2 (45)	9.8 (104)	38.6 (411)	31.6 (337)	15.9 (169)	3.451	1.007	−0.308	−0.156	0.257
Socio-economic issues (SEI)	Shut down or lockdown of regular activities is a good decision to reduce the chance of infection from COVID-19	SEI1	1.5 (16)	1.2 (13)	4.8 (51)	27.4 (292)	65.1 (694)	4.534	0.774	−2.186	5.877	0.341
	Shut down or lockdown or social distancing will have an economic and social impact in the future	SEI2	1.1 (12)	1.4 (15)	6.5 (69)	27.2 (290)	63.8 (680)	4.511	0.774	−1.935	4.453	0.486
	Formal and informal business will be hampered	SEI3	0.7 (7)	1 (11)	6.3 (67)	30.9 (329)	61.2 (652)	4.508	0.719	−1.701	3.719	0.513
	Poor people living hand-to-mouth will be severely affected	SEI4	0.8 (9)	0.6 (6)	3.1 (33)	9.9 (106)	85.6 (912)	4.788	0.604	−3.639	15.449	0.525
	Most of the poor people living in urban areas have to leave due to not having any options for income	SEI5	1.4 15	3 (32)	8.6 (92)	26.1 (278)	60.9 (649)	4.420	0.875	−1.7	2.797	0.430
	Many people will lose their livelihood/ jobs at this time	SEI6	1.3 (14)	2.5 (27)	9.6 (102)	31.8 (339)	54.8 (584)	4.362	0.856	−1.515	2.416	0.490
	There will be a lower supply of basic goods/ products for daily use	SEI7	2 (21)	5.6 (60)	12.8 (136)	36.8 (392)	42.9 (457)	4.130	0.971	−1.149	0.956	0.412
	Prices of the most basic products will be higher than usual	SEI8	1 (11)	4 (43)	10.7 (114)	33.5 (357)	50.8 (541)	4.289	0.887	−1.303	1.46	0.401
	Poor people will suffer from food and nutritional deficiency	SEI9	0.9 (10)	1.2 (13)	4 (43)	24.4 (260)	69.4 (740)	4.601	0.712	−2.301	6.604	0.531
	The formal education system will be hampered	SEI10	1.7 (18)	2.3 (25)	9.5 (101)	29.4 (313)	57.1 (609)	4.379	0.877	−1.628	2.756	0.448
	There is a chance of social conflict due to this outbreak	SEI11	3.8 (40)	6.4 (68)	20.6 (220)	34.3 (366)	34.9 (372)	3.902	1.068	−0.863	0.201	0.408
Immediate emerging issues (IEI)	There is a chance of community transmission of COVID-19 in Bangladesh	IEI1	0.9 10	1.2 (13)	12.2 (130)	29.3 (312)	56.4 (601)	4.389	0.817	−1.373	1.897	0.459
	A huge number of people will be infected	IEI2	1.2 (13)	3.5 (37)	16.9 (180)	30.1 (321)	48.3 (515)	4.208	0.926	−1.056	0.639	0.466
	There is a chance of not detecting most of the infected patients due to the lack of health facilities, which leads to undermining the number of actual infected cases	IEI3	0.9 (10)	1.2 (13)	7.9 (84)	21.1 (225)	68.9 (734)	4.557	0.769	−1.99	4.303	0.508
	There is a chance of increasing the number of deaths by not having proper health facilities	IEI4	0.8 (9)	1.3 (14)	5.7 (61)	25 (266)	67.2 (716)	4.563	0.736	−2.04	4.96	0.594
	Lack of bio-medical waste management facilities in the hospitals will create environmental transmission	IEI5	0.7 (7)	1.1 (12)	6.7 (71)	30 (320)	61.5 (656)	4.507	0.728	−1.698	3.573	0.583
	Many people will be psychosocially shocked due to this outbreak	IEI6	0.6 (6)	2.1 (22)	12.2 (130)	38.7 (413)	46.4 (495)	4.284	0.800	−1.064	1.111	0.498
	The government will lose its trust from the people	IEI7	3.8 (41)	7.4 (79)	27.1 (289)	25.2 (269)	36.4 (388)	3.829	1.118	−0.642	−0.362	0.341
Enduring emerging issues (EEI)	There is a chance of a disaster like a flood, cyclone, or landslide in 2020 considering the climate change vulnerability of Bangladesh	EEI1	2.6 (28)	7.1 (76)	32.8 (350)	32.6 (348)	24.8 (264)	3.698	1.004	−0.417	−0.254	0.329
	If any disaster (flood, cyclone, landslide) occurs after/during the COVID-19 situation then it will create a double burden to the country	EEI2	0.4 (4)	1.2 (13)	5.5 (59)	18.8 (200)	74.1 (790)	4.650	0.676	−2.202	5.301	0.544
	There is a chance of severe food scarcity due to these events (COVID-19 + Disasters) in the country	EEI3	0.6 (6)	2.7 (29)	13.2 (141)	33.1 (353)	50.4 (537)	4.300	0.840	−1.116	0.89	0.459
	High possibility of huge economical loss	EEI4	0.5 (5)	0.7 (7)	5 (53)	27.2 (290)	66.7 (711)	4.590	0.661	−1.875	4.59	0.555
	High possibility of increasing the poverty level	EEI5	0.5 (5)	2.3 (25)	8.5 (91)	31 (330)	57.7 (615)	4.431	0.783	−1.449	2.065	0.574
	High possibility of severe socio-economic and health crisis	EEI6	0.5 (5)	1.5 (16)	6.3 (67)	32.6 (347)	59.2 (631)	4.485	0.723	−1.57	3.004	0.602

There was a limitation of the rapid assessment on the public-perception on the psychosocial and socio-economic crisis in Bangladesh due to the COVID-19 pandemic. As the study was conducted during the lockdown period, it was not possible to reach to general people physically. Therefore, we had to keep our samples limited to internet users only. There are more than 95 million mobile internet users in Bangladesh and, as a youth-dividend country, the majority of the mobile internet users are young educated people.

### Data Analysis

The descriptive statistics [e.g., frequencies, percentages, and *T*-test (data provided in Supplementary Tables)] were employed to understand respondents' characteristics. An investigation of psychometric characteristics was included in the Classical Test Theory (CTT) analysis. A set of statistical techniques, including linear regression analysis (LRA), principal component analysis (PCA), and hierarchical cluster analysis (CA), were applied to explore the association between the items. PCA is a data reduction tool that demonstrates each potentiality of parameters and their confidence level in large sample datasets. Before conducting the PCA, Kaiser-Maier-Olkin (KMO) and Bartlett's sphericity tests were applied to confirm the necessity of this analysis. The results of the KMO at >0.5 (the KMO value was 0.931 in this work) and the significance of Bartlett's sphericity test at *p* < 0.01 supported our datasets to be fitted for the PCA (16). The number of factors chosen was based on the Kaiser's principle, where the only factors with eigenvalues>1.0 were considered. Cronbach's alpha was employed to test the consistency and reliability of the factor loadings in this study. Cronbach's alpha values at >0.06 (the Cronbach's alpha value was 0.896) are regarded to be suitable in social science research (17). The CA is a crucial means of detecting associations among many psychosocial and environmental parameters. CA assists to demarcate a population into various groups based on the same feature of a set of the dataset that may reveal causes, effects, and/or the source of any unidentified relationships among the items. Furthermore, hierarchical clustering was used to determine the probable number of clusters. Statistical Package for the Social Sciences (SPSS) v. 25.0 was used for the analysis of the datasets.

### Ethics Statement

The consent of the respondents was taken before the survey, and their anonymity was guaranteed. All the participants were informed about the specific objective of this study before proceeding to the questionnaire. Participants were able to complete the survey only once and could terminate the survey at any time they desired. Anonymity and confidentiality of the data were ensured. Formal ethical permission of this study was taken from the respective authority.

## Results

### Demographics Information

A total of 1,066 (=n) responses were recorded in this study. The proportion of male to female respondents was 3:2 [males (*n* = 661; 61.5%) and females (*n* = 405; 38.5%)]. The composition of age groups of the respondents was as follows: 75.2% (18–30 years old), 16.7% (31–40 years old), 6.7% (41–50 years old), 1.1% (51–60 years old), and 0.3% (>60 years old). The average age of the respondents was 27.80 years (SD ± 10.05). On average, the respondents had 12.5 years of formal education (SD ± 8.1). 60% of the youth group were mostly students or at the brink of finishing their studies. The remaining 40% of the respondents were from various professions, including doctors and healthcare workers, civil service officials, non-government officials (NGOs), teachers and scholars, policymakers, researchers, and businessmen.

### A Descriptive Overview of the People's Perception

The descriptive statistics containing the 46 statements are shown in Table 1. The category of statements were grouped as follows: Mental health condition (MH) comprised five statements (MH1-5), the healthcare system of Bangladesh (HSB) comprised ten statements (HSB1-10), the governance and political issues (GPI) comprised 7 statements (GPI1-7), the socio-economic issues comprised 11 statements (SEI1-11), the immediate emerging issues comprised 7 statements (IEI1-7), and for enduring emerging issues 6 statements were considered (EEI1-6). In the following section of Mental Health Status, Healthcare System, Governance and Political Perspective, Socio-Economic Aspects, and Emerging Issues, we have discussed the descriptive statistics.

#### Mental Health Status

In the statement of “*I am afraid of the recent outbreak of coronavirus in Bangladesh”* (MH1) 46.2% of the respondents strongly agreed, followed with a mean of 4.15 ± 1.01. In the second statement (MH2), “*I am afraid of getting infected with coronavirus”* the difference among strongly agreed (32.7%) and agreed (33.5%) statement with a mean value of 3.89 ± 1.08. For statement three, 46.5% of the respondents strongly agreed to the (MH3) “*I am afraid of losing my life or my relatives' life due to this outbreak”* with a mean value of 4.08 ± 1.08. In the fourth statement (MH4), “*All the news of infection and deaths from COVID-19 in different media is increasing my fear”* 43.5% of the respondent strongly agreed, with a mean response of 4.07 ± 1.05. 51.7% of the respondents strongly agreed with the fifth statement (MH5) “*It makes me uncomfortable to be detached from regular activities due to lockdown”* with a mean value of 4.19 ± 1.05.

#### Healthcare System

62% of the respondents strongly agreed to the statement that the healthcare system of Bangladesh is fragile and unable to deal with the recent outbreak of COVID-19 (HSB1), with a mean value of 4.36 ± 1.01. For the second statement, 68% of respondents with a mean value of 4.51 ± 0.87 strongly agreed that “*a huge population is a pressure to the existing healthcare system to deal with COVID-19”* (HSB2). 67% of the respondents with a mean value of 4.55 ± 0.776 strongly agreed that “*there is a lack of awareness of basic healthcare issues in most of the citizens of Bangladesh”* (HSB3). Moreover, 52% of the respondents with a mean value of 4.22 ± 1.0 strongly agreed that there is “*a lack of trained doctors and healthcare professionals to deal with the COVID-19”* (HSB4). With a mean value of 4.64 ± 0.73, 75.4% of the respondents strongly agreed that “*the lack of healthcare facilities will be unable to combat the COVID-19 outbreak in Bangladesh”* (HSB5). Again, 73% of respondent with a mean of 4.6 ± 0.77 strongly agreed with “*the lack of healthcare infrastructure to deal with COVID-19”* (HSB6). For statement seven, 68.5% of respondents with a mean value of 4.56 ± 0.734 strongly agreed that “*there is a severe lack of bio-medical and hospital waste management facilities in Bangladesh”* (HSB7). Moreover, 82% of respondents with a mean value of 4.72 ± 0.71 strongly agreed that “*there is a lack of COVID-19 testing facility in Bangladesh”* (HSB8). 49.2% of respondents (4.05 ± 1.86) strongly agreed that “*the budget is inadequate or there is a lack of financial support to respond to this outbreak”* (HSB9). Finally, 75.8% of respondents with a mean value of 4.64 ± 0.74 strongly agreed that “*most of the poor people will not have access to the existing healthcare facilities if they are infected with COVID-19”* (HSB10).

#### Governance and Political Perspective

Regarding the statement of “*the Bangladesh government can deal with this outbreak”* (GPI1), the public opinion did not vary significantly with a mean value of 2.50 ± 1.28. Similar responses were also found in response to “*the Government is taking this outbreak seriously”* (GPI2) with a mean value of 2.76 ± 1.26 and “*the Government is taking proper decisions at the right time”* (GPI3) with a mean value of 2.27 ± 1.19. 68.6% of respondents strongly agreed that “*the Government needs support from the general public to reduce the impact of COVID-19”* (GPI5) with a mean value of 4.56 ± 0.77 and that “*the Government needs to formulate a policy and action plan and implement it immediately”* (GPI6) with a mean value of 4.67 ± 0.69. About 31.6% of respondents agreed that “*developed nations are going to support Bangladesh in response to COVID-19”* (GPI7) with a mean value of 3.45 ± 1.0.

#### Socio-Economic Aspects

Nearly 61–65% of respondents strongly agreed that “*the shut down or lockdown of regular activities was a good decision to reduce the chance of infection of COVID-19”* (SEI1) (mean 4.53 ± 0.77), “*this will have an economic and social impact in the future”* (SEI2) (mean 4.51 ± 0.77), and that “*both formal and informal businesses will be hampered” (SEI3)* (mean 4.5 ± 0.71). For the fourth statement, 85.6% of respondents strongly agreed that “*poor people living off daily wages will be severely affected”* (SEI4) with a mean of 4.78 ± 0.60, while 60.5% strongly agreed that “*most of the poor people living in urban areas have to leave the city due to not having any options for income”* (SEI5) (mean 4.42 ± 0.87). 54.8% (mean 4.36 ± 0.85) of the respondents agreed that “*many people will lose their livelihood/ jobs at this time”* (SEI6). A further 42.9% (mean 4.13 ± 0.97) strongly agreed that “*there will be a reduced supply of basic goods/ products for daily use”* (SEI7) and 50.8% (mean 4.28 ± 0.89) strongly agreed that “*there was or will be increased prices for basic products”* (SEI8). Consequently, “*poor people will suffer food and nutritional deficiency*” (SEI9) was strongly agreed with by 69.4% respondents (mean value of 4.6 ± 0.712). “*The shutdown of education institutes will hamper those currently receiving formal education”* (SEI10), to which 57% respondents strongly agreed (mean value of 4.38 ± 0.88). For “*If there is a chance of social conflict due to this outbreak”* (SEI11), the mean response was 3.9 ± 1.06.

#### Emerging Issues

56.4% (mean 4.39 ± 0.82) of respondents strongly considered that “*there is a chance of community transmission of COVID-19 in Bangladesh”* (IEI1) and that “*a huge number of people will be infected”* (IEI2) with a mean value of 4.208 ± 0.93. Moreover, 69% of the respondents strongly agreed (mean value 4.56 ± 0.74) that “*there is a chance that many infected patients will not be detected due to a lack of testing facilities and this will not show the actual number of infected cases*” (IEI3). Approximately 61–67% of the respondents strongly agreed that “*there is a chance of an increasing numbers of deaths from infection due to a lack of proper health facilities*” (IEI4) with a mean value of 4.56 ± 0.74. “*A lack of bio-medical waste management facilities in the hospitals will create further transmission”* (IEI5) received a mean value of 4.50 ± 0.73. For the sixth statement, 46.4% of respondents (mean value of 4.28 ± 0.88) strongly agreed that “*there will be many people psychosocially shocked due to this outbreak”* (IEI6) and that “*the general public will lose trust in the government”* (IEI7) was strongly agreed with by 36.4% respondents with a mean value of 3.83 ± 1.12.

We have considered emerging enduring issues (EEI), such as potential natural calamities and infectious disease outbreaks, as the monsoon season is approaching. Six statements were considered for enduring emerging issues (EEI1-6). Regarding the statement that “*there is a chance of a disaster such as a flood, cyclone, or drought in 2020 considering the vulnerability of Bangladesh to climate change”* (EEI1), there was a mean response of 3.7 ± 1.0. But the statement “*if any disaster (flood, cyclone, landslide) occurs after/during COVID-19, the situation will create a double burden to the country”* (EEI2) was strongly agreed with by 74% of respondents with a mean of 4.65 ± 0.68. 50.4% of respondents agreed with a mean of 4.3 ± 0.84 that “*there is a chance of severe food scarcity in the country due to these events (COVID-19* + *Disasters)”* (EEI3). A strong agreement from participants (varied from 50 to 66%) was observed for the statements: “*there is a high possibility of huge economical loss”* (EEI4) with a mean value of 4.59 ± 0.66, “*there is a high possibility of increasing poverty level”* (EEI5) with a mean value of 4.43 ± 0.78, and “*there is a high possibility of severe socio-economic and health crisis”* (EEI6) with a mean value of 4.48 ± 0.72.

### Results From Regression Analysis

#### The Association of Affected Psychosocial Wellbeing and the Fragile Healthcare System During COVID-19 Outbreak

From the regression analysis, among the 45 variables, only five variables showed statistically significant associations with the fragile healthcare system of Bangladesh (HSB1) to deal with the recent outbreak of COVID-19 in the country (Table 2). HSB2, HSB5, and IEI1 statistically pose a significant positive effect on the fragile healthcare system of Bangladesh (*p* < 0.01). This relationship implies that a huge population and a lack of healthcare facilities are contributing to the community transmission of COVID-19 in Bangladesh. The presence of community transmission in Bangladesh within a short time is present as predicted by the IEDCR, who announced a mild-level community transmission possibility in Bangladesh on 1st April 2020 in their press release (9). This assumption is further validated by the number of deaths from COVID-19 reported in the news, after the announcement of the partial lockdown, and the opening of RMG factories from 25 April 2020. The number of COVID-19 patients increased significantly in industrial zones. There was also a positive significant association between the fear of the COVID-19 outbreak (MH1) with the struggling healthcare system (*p* < 0.05). Also, the negative association between HSB1 and government political decision GPI1 (*p* < 0.05) reveals that the Government is unable to make proper decisions at the right time due to the poor governance in the existing healthcare system.

**Table 2:** Multiple linear regression models for selected statements using perceptions as independent variables (*n* = 1,066).

**Perception statement (multiple *R*^2^ value)**	**Constant of model**	**Significant (1% and 5% level) regression coefficients for independent variables (standard error of regression coefficients)**									**Standard error of regression model (ANOVA, *F*-statistic)**
MH1 (0.472)	0.297	+0.314 MH2 (0.03)	+0.14 MH3 (0.03)	+0.181 MH4 (0.026)	+0.053 HSB1 (0.026)	+0.087 HSB8 (0.046)	+0.049 GPI7 (0.025)	−0.075 SEI10 (0.032)	+0.104 IEI1 (0.036)	0.117 IEI2 (0.033)	0.75162 (20.264)
HSB1 (0.252)	0.514	0.075 MH1 (0.037)	0.125 HSB2 (0.036)	0.296 HSB5 (0.058)	−0.076 GPI3 (0.032)	0.118 IEI1 (0.043)					0.89242 (7.619)
GPI1 (0.242)	1.847	−0.083 HSB4 (0.041)	−0.206 HSB9 (0.033)	0.253 GPI2 (0.039)	0.149 GPI3 (0.04)	0.127 GPI7 (0.038)	0.224 SEI2 (0.062)	−0.225 SEI3 (0.072)	0.084 SEI11 (0.04)		1.14184 (7.237)
SEI2 (0.479)	−0.238	0.054 MH3 (0.023)	0.085 HSB5 (0.038)	0.056 GPI1 (0.016)	0.045 GPI3 (0.02)	0.517 SEI3 (0.032)	0.098 SEI4 (0.04)	0.049 SEI8 (0.025)	0.048 SEI10 (0.025)	0.084 EEI6 (0.038)	0.57089 (20.859)
IEI1 (0.396)	0.66	0.078 MH1 (0.027)	0.077 MH3 (0.026)	0.062 HSB1 (0.023)	0.169 HSB7 (0.039)	0.082 SEI6 (0.031)	0.077 SEI11 (0.023)	0.246 IEI2 (0.028)	0.178 IEI3 (0.036)		0.64904 (14.864)
EEI2 (0.428)	0.495	−0.047 MH2 (0.022)	0.046 HSB2 (0.021)	0.09 SEI4 (0.036)	0.11 SEI9 (0.031)	0.088 IEI5 (0.031)	0.085 EEI1 (0.018)	0.112 EEI3 (0.024)	0.178 EEI4 (0.037)	0.07 EEI6 (0.034)	0.52213 (16.989)

#### The Affected Psychosocial Wellbeing and Socio-Economic Fear of COVID-19 and the Government's Decision to Lockdown

The results of linear regression showed that among the 45 variables, only 10 variables showed statistically significant associations with fear of the COVID-19 outbreak (Table 2). For instance, mental health variables MH2, MH3, and MH4 statistically pose a significant positive effect on fear of the COVID-19 outbreak (*p* < 0.01). On the other hand, there is a statistically positive association between fear of the COVID-19 outbreak (*p* < 0.05)and the healthcare system in Bangladesh (HSB1 and HSB8), due to the lack of testing facilities and a fragile healthcare system contributing to the fear that has been experienced due to the COVID-19 pandemic in Bangladesh. The socioeconomic issues (SEI 10) and immediate emerging issues (IEI2) have a statistically significant positive impact (*p* < 0.01), e.g., obstruction to the formal education system, and the potentiality of a huge number of people becoming infected may contribute to the fear development of the COVID-19 outbreak in this country. There was also a positive significant association between the chance of community transmission of COVID-19 for immediate emerging issues (IEI1) with fear of the COVID-19 outbreak (*p* < 0.05).

Results from the regression analysis further showed eight variables have a significant statistical association with the governance and political capacity to deal with the COVID-19 outbreak in Bangladesh (GPI1). A significant positive association was found among the governance and political issues (GPI1 with GPI2 and GPI3) and socioeconomic issues (SEI2) (*p* < 0.01), implying that the government's decision to lockdown activities was at the proper time and has enhanced the people's perception of the capacity of Government to deal with the COVID-19 outbreak (Table 2).

#### The Potential Arising of Social Conflicts From COVID-19 and Governance and Political Association

However, the negative association between governance and political issues (GPI1) and the healthcare system of Bangladesh (HSB9) (*p* < 0.01) shows that a perceived lack of budget created a gap in the response to COVID-19 (Table 2). Moreover, a negative association of governance and political issues (GPI1) with the healthcare system of Bangladesh (HSB4) and socioeconomic issues (SEI3) (*p* < 0.05) shows a perceived lack of trained doctors and healthcare professionals, and that a hampering of formal and informal business activities are reducing the government's capacity to deal with the COVID-19 outbreak. Nevertheless, a positive association of governance and political issues GPI1 with socioeconomic issues SEI11 (*p* < 0.05) and governance and political issues GPI7 (*p* < 0.01) shows that there is a perceived possibility of social conflict due to this outbreak if not managed properly, and that the Bangladesh Government will need support from developed nations and allied forces to deal with this outbreak. It should be mentioned here that containment, risk mitigation, and suppression plans must be as inclusive as possible or risk undermining response efforts.

#### The Potential Socioeconomic Crisis of the COVID-19 Outbreak and the Suffering Poor Communities

The regression analysis showed that, among the 45 variables, nine showed a significant statistical association with the future impacts of implementing lockdown and social-distancing activities (SEI2). A significant positive association of socioeconomic issues (SEI2) with governance and political issues (GPI1) and socioeconomic issues (SEI3) (*p* < 0.01) shows that the Government took the right decision by shutting down regular activities and implementing the social distancing approach (Table 2). But due to this initiative, the formal and informal business sectors and the economy will be hampered. Again, a positive association of socioeconomic issues (SEI2) with mental health (MH3) and healthcare services (HSB5) (*p* < 0.05) reveals that this decision of shutting down normal activities was imposed due to the fear of losing lives due to COVID-19 and having a lack of healthcare facilities. However, a positive association of socioeconomic issues SEI2 with SEI4, SEI8, SEI10, and enduring emerging issues EEI6 (*p* < 0.05) shows that due to this shut down poor people will be severely affected, the price of the basic products will increase, the formal education system will be hampered, and the possibility of severe socio-economic and health crises will increase.

#### Other Infectious Disease Risk Management During COVID-19 Outbreak

In the regression analysis, eight variables are statistically associated with the possibility of community transmission of COVID-19 (IEI1). A significant positive association between mental health variables (MH1, MH3), healthcare system variables (HSB1, HSB7), Socioeconomic variables (SEI6, SEI11), and immediate emerging issues (IEI2, IEI3) (*p* < 0.01) reveals that community transmission will increase the number of infected people which will create further fear and mental pressure of others of losing their lives due to COVID-19 infection (Table 2). The fragile healthcare system of Bangladesh will be unable to detect most of the infected patients due to a lack of health facilities, which leads to undermining the actual infected cases. As of the last day of the survey for this study on 30 March 2020, the testing rate of COVID-19 was at its lowest in Bangladesh compared to the other similar countries (10 people/ 1 million). However, as the laboratories increased, the number of testing has increased along with this, with 878 people/1 million. This is still inadequate compared to the population density. Also, the inadequate disposal method of COVID-19 hospital bio-medical waste management and associated facilities could increase community transmission. Subsequently, due to the community transmission of COVID-19, many people will lose their lives and livelihoods, which might lead to creating social conflict, as a worst-case scenario.

#### Combating Environmental and Climate-Induced Natural Disaster Risks During the COVID-19 Outbreak

The regression analysis further identified nine variables that are significantly associated with the possibility of climate-induced extreme natural events (flood, cyclone, landslides, etc.) occurring during/after the COVID-19 pandemic. The pandemic along with natural disasters may create a double burden to the country due to enduring emerging issues (EEI2). The positive association between EEI2, SEI9, IEI5, EEI1, EEI3, and EEI4 (*p* < 0.01) shows that there is a perceived possibility of a climate-change-induced disaster after the COVID-19 situation which would create severe food insecurity (Table 2). Poor people will suffer most from food and nutritional deficiency and the country will face enormous economic loss. Also, after the COVID-19 situation, a lack of bio-medical and solid waste management will add more problems. Moreover, a positive association between EEI2, HSB2, and EEI6 reveals that, after the COVID-19 emergency, existing poverty will create further socio-economic and health crises.

### Overall Relationship Assessment Among the Variables From CTT, PCA, and CA

CTT and PCA revealed a confidence level of controlling factors in Bangladesh during the COVID-19 outbreak and how these components are correlated to the psychosocial, socio-economic, and environmental crisis components (Tables 1, 3). Cluster analysis (CA) further detected the total status of regional variations, and how socio-economic and environmental crises influences psychosocial development (**Figure 3**).

**Table 3:** Varimax rotated principal components.

	**PC1**	**PC2**	**PC3**	**PC4**	**PC5**	**PC6**	**PC7**	**PC8**	**PC9**
MH1	0.122	−0.034	0.25	0.03	0.746	−0.048	0.065	0.049	0.055
MH2	0.041	0.051	0.162	0.094	0.832	−0.041	0.05	−0.047	−0.061
MH3	0.064	0.07	0.172	0.112	0.764	−0.071	0.052	−0.071	−0.031
MH4	0.103	0.088	0.005	0.029	0.613	0.012	0.106	0.118	0.32
MH5	0.171	0.123	−0.036	−0.002	0.41	0.126	0.067	0.313	0.213
HSB1	0.347	−0.047	0.168	0.055	0.106	−0.173	0.098	0.3	0.383
HSB2	0.334	0.031	−0.088	0.204	0.126	−0.024	0.353	0.12	0.43
HSB3	0.459	0.187	0.052	0.076	0.162	−0.123	0.244	0.03	0.197
HSB4	0.637	0.018	−0.008	0.105	0.024	0.003	−0.031	0.009	0.065
HSB5	0.743	0.065	0.218	0.071	0.073	−0.058	0.165	0.124	0.071
HSB6	0.746	0.056	0.213	0.122	0.086	−0.028	0.057	0.072	0.045
HSB7	0.682	0.089	0.216	0.108	0.034	−0.051	0.147	0.166	0.119
HSB8	0.689	0.149	0.221	0.031	0.026	−0.013	0.231	0.034	−0.027
HSB9	0.54	0.263	−0.058	0.166	0.137	0.004	−0.172	−0.143	−0.081
HSB10	0.5	0.292	0.26	0.135	0.095	−0.052	0.286	−0.072	−0.026
GPI1	−0.162	−0.001	0.065	−0.183	−0.061	0.571	0.029	0.062	0.078
GPI2	−0.017	−0.05	−0.186	−0.012	−0.056	0.787	0.111	0.1	−0.059
GPI3	−0.074	−0.07	−0.158	0.012	−0.05	0.783	−0.044	0.026	−0.039
GPI4	0.018	−0.041	−0.096	0.035	−0.001	0.698	0.078	0.001	−0.047
GPI5	0.205	0.047	0.175	0.098	0.032	0.214	0.651	0.133	0.006
GPI6	0.233	0.211	0.298	0.033	0.033	0.044	0.627	0.011	0.039
GPI7	0.056	0.156	0.205	0.068	0.151	0.432	0.085	−0.246	0.088
SEI1	0.053	0.086	0.071	0.068	0.152	0.085	0.574	0.09	0.063
SEI2	0.092	0.336	0.135	0.211	0.035	0.115	0.157	0.636	−0.018
SEI3	0.108	0.37	0.103	0.267	0.035	0	0.225	0.637	−0.024
SEI4	0.115	0.493	0.107	0.188	0.105	−0.045	0.397	0.291	−0.136
SEI5	0.109	0.606	0.051	0.124	0.052	0.055	0.131	0.111	−0.024
SEI6	0.059	0.657	0.192	0.171	0.073	−0.036	0.05	0.164	0.002
SEI7	0.106	0.702	0.014	0.18	0.042	−0.024	−0.035	−0.108	0.152
SEI8	0.106	0.636	0.014	0.12	−0.003	−0.151	0.126	0.024	0.151
SEI9	0.132	0.638	0.107	0.205	0.02	−0.057	0.322	0.115	−0.054
SEI10	0.072	0.418	0.158	0.261	0.001	0.151	0.033	0.317	0.089
SEI11	0.137	0.548	0.208	0.058	0.101	0.059	−0.29	0.201	0.229
IEI1	0.151	0.058	0.663	0.111	0.153	−0.031	0.025	0.177	0.093
IEI2	0.126	0.122	0.655	0.164	0.291	−0.078	0.057	−0.062	−0.036
IEI3	0.263	0.108	0.591	0.145	0.084	−0.198	0.261	0.125	−0.007
IEI4	0.267	0.111	0.642	0.253	0.135	−0.081	0.28	0.039	−0.001
IEI5	0.239	0.121	0.546	0.244	0.151	−0.03	0.239	0.068	0.156
IEI6	0.064	0.277	0.383	0.141	0.078	0.087	0.256	−0.039	0.424
IEI7	0.104	0.281	0.453	0.033	0.04	−0.251	−0.083	−0.034	0.37
EEI1	0.041	0.157	0.114	0.345	0.131	0.043	−0.085	−0.107	0.519
EEI2	0.182	0.146	0.261	0.541	0.043	−0.061	0.236	0.102	0.181
EEI3	0.157	0.227	0.068	0.683	0.082	0.008	0.002	−0.124	0.187
EEI4	0.154	0.208	0.119	0.75	0.093	−0.041	0.093	0.244	0.004
EEI5	0.119	0.321	0.194	0.686	0.09	−0.027	0.067	0.16	0.021
EEI6	0.171	0.27	0.274	0.659	0.029	−0.059	0.11	0.191	0.073
**Eigenvalues**	4.125	3.95	3.31	3.124	2.771	2.577	2.44	1.722	1.41
**% of Variance**	8.967	8.587	7.196	6.792	6.023	5.603	5.304	3.743	3.064
**Cumulative %**	8.967	17.555	24.75	31.543	37.566	43.169	48.473	52.215	55.28

#### Results From CTT and PCA

From the CTT analysis, according to the corrected inter-item correlation analysis, among 46 variables, four variables have low corrected item-total correlations (i.e., the ability of the government to deal the outbreak, −0.054; seriousness of the government, −0.011; government is taking a proper decision, −0.078; and other sectoral involvement to COVID-19, −0.04). The remaining 42 variables in the scale had an acceptable corrected item-total correlation (0.257 to 0.602) and the Cronbach's alpha (0.896) was acceptable.

**Figure 2:**
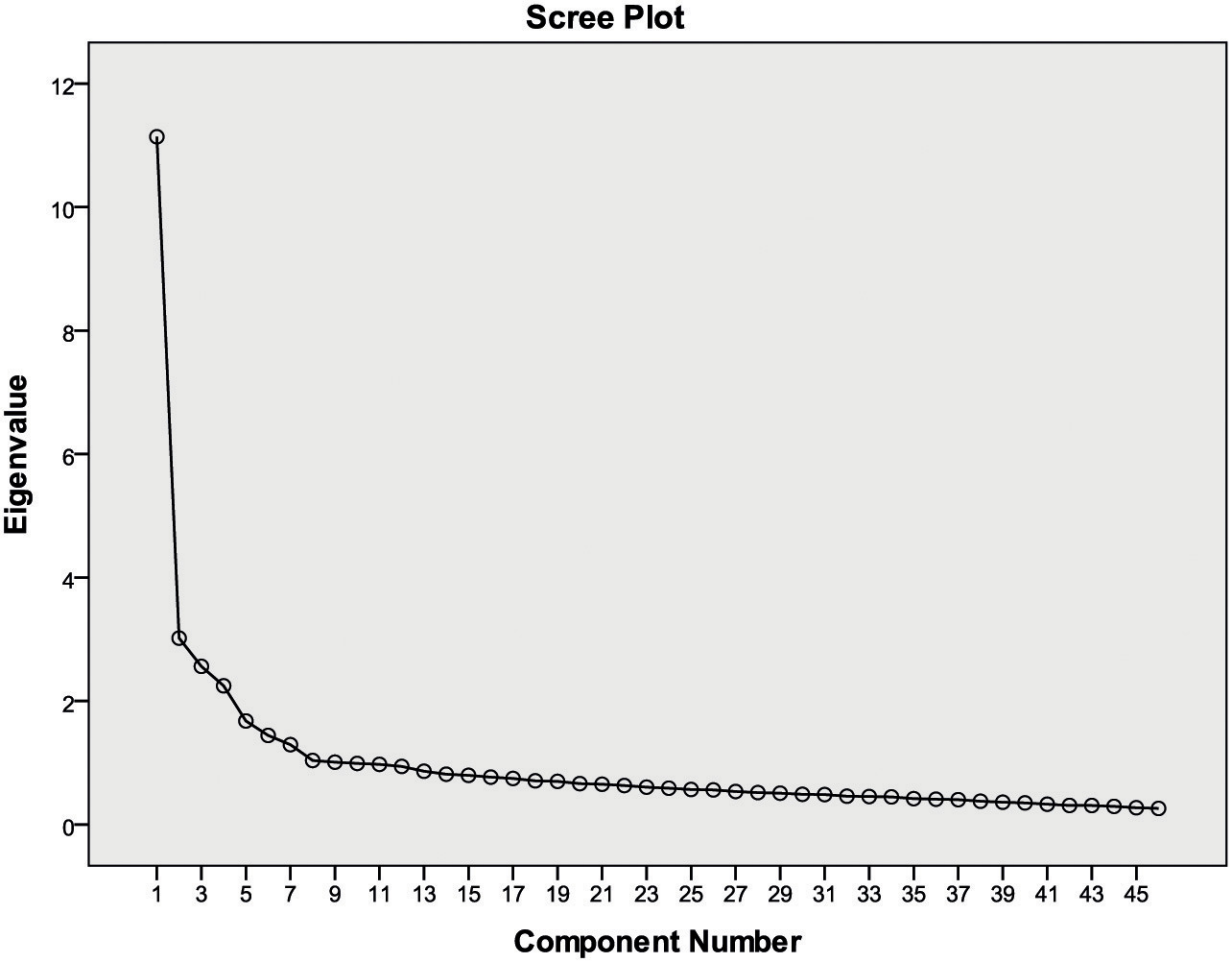
Scree plots of the eigenvalues of PCA.

From PCA, nine principal components (PCs) were originally based on standard eigenvalues (surpassed 1) that extracted 55.28% of the total variance as displayed in Table 3. The scree plot was adopted to detect the number of PCs to be retained to provide insight into the underlying variable internal structure (Figure 2). The loading scores were demarcated into three groups of weak (0.50–0.30), moderate (0.75–0.50), and strong (>0.75) (18–20).

The PC1 (First) showed 8.967% of variance as it encompassed a confidence level of weak positive loading of the healthcare system in Bangladesh (HSB1-3: 0.334–0.459); with results being moderate positively loaded for the healthcare system in Bangladesh (HSB4-10: 0.50–0.746). The PC2 (Second) indicated 8.587% of the variance and was loaded with moderate positive loading for socio-economic issues (SEI5-9: 0.606–0.702 and SEI11: 0.548) and weak positively loaded for socio-economic issues (SEI2-4: 0.336–0.493 and SEI10: 0.418).

The PC3 (Third) showed 7.196% of the variance and was moderate positively loaded for immediate emerging issues IEI1-5 (0.546–0.665). The PC4 (Four) indicated 6.792% of the variance, and was loaded with a significant level of strong positive loadings for immediate emerging issues IEI4 (0.751); results were moderate positively loaded for immediate emerging issues IEI2-3 (0.541–0.683) and immediate emerging issues IEI5-6: 0.659–0.686), and were weak positively loaded for immediate emerging issues IEI1 (0.345).

The PC5 (Five) and PC6 (Six) indicated 6.023 and 5.603% of the total variances, and loaded a significant level of strong positive loading for mental health issues MHI2-3 (0.764–0.832) and government and political issues GPI2-3(0.783–0.787); results were moderate positively loaded for mental health issues MHI1 (0.746), MHI4 (0.613), government and political issues GPI1 (0.571), and GPI4 (0.698). Results were weak positively loaded for mental health issues MHI5 (0.41) and government and political issues GPI7 (0.574).

The PC7 (Seven), PC8 (eight), and PC9 (nine) showed 5.304, 3.743, and 3.064% of the total variances and were moderate positively loaded for government and political issues GPI5-6 (0.627–0.651), socioeconomic issues SEI1 (0.574), SEI2-3 (0.636–0.637), and immediate emerging issues (IEI1:0.519); results were weak positively loaded for socio-economic issues SEI4 (0.397), SEI9-10 (0.317–0.322), healthcare sector of Bangladesh HSB1-2 (0.383–0.430), mental health issues MHI5 (0.313), and immediate emerging issues IEI6-7(0.370–0.424).

#### Results From the Cluster Analysis (CA)

**Figure 3:**
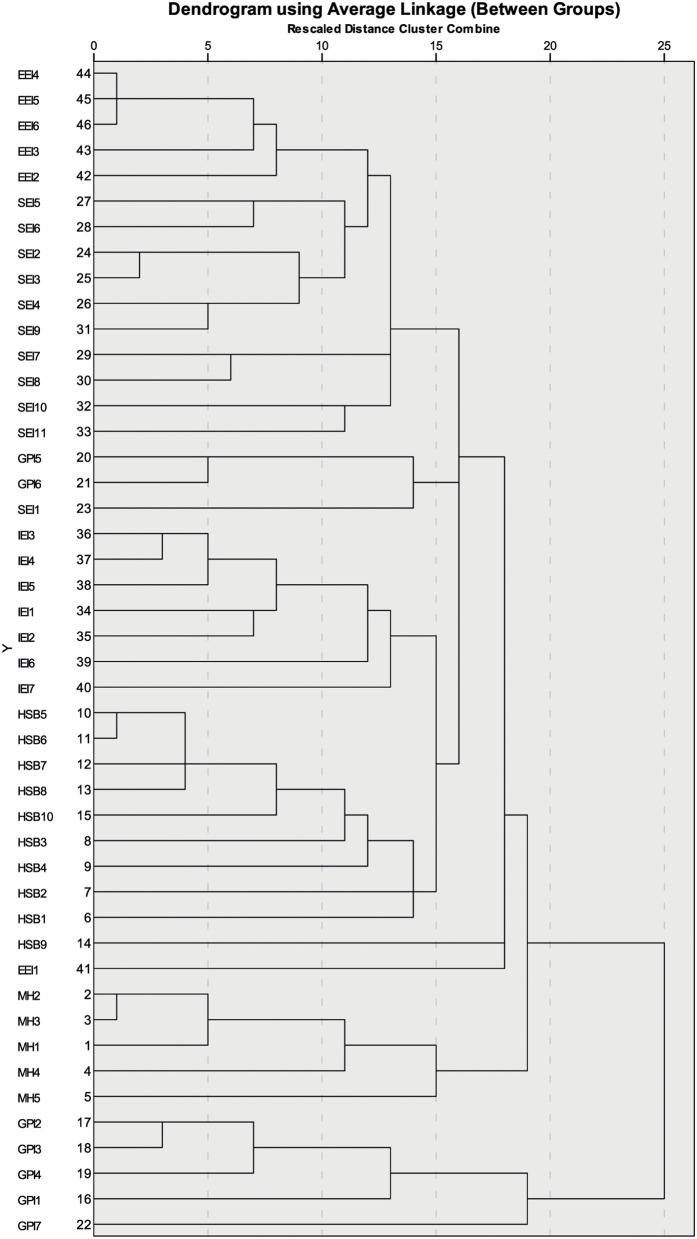
Dendrogram showing the clustering of people's perceptions on the COVID-19 outbreak in Bangladesh.

In the CA all the parameters were classified into four major groups: cluster-1(C1), cluster-2 (C2), cluster-3(C3), and cluster-4(C4) (Figure 3). C1 was composed of two sub-clusters of C1-A and C1-B; C1-A was composed of issues surrounding an increase in the number of deaths due to not having proper health facilities, a lack of bio-medical waste management facilities in Bangladesh that will create more problems, many people experiencing psychosocial issues due to this outbreak, with a large number of people becoming infected, and there being a chance of not detecting most of the infected patients due to the lack of health facilities leading to undervaluing the actual infected cases (IEI4-6, IEI2-3). C1-B was composed of socio-economic issues that may lead to poor people suffering from a lack of food, thereby leading to nutritional deficiency (SEI2-6 and SEI9). C2 consists of socio-economic issues (SEI7-11). C3 consisted of three sub-clusters of C3-A, C3-B, and C3-C. C3-A covered governance and political issues GPI5-6, and socio-economic issues (SEI1). C3-B consisted of immediate emerging issues IEI1-7, while C3-C was composed of issues related to the healthcare system in Bangladesh (HSB1-10). Cluster-4 consisted of three sub-clusters of the C4-A health system in Bangladesh and immediate emerging issues (HBS9, IEI1), C4-B covered mental health issues (MHI1-5), and C4-C contained governance and political issues (GPI1-4 and GPI7).

## Discussion

### Societal Fear and Anxiety Over COVID-19 in Bangladesh

This perception-based study tried to visualize the psychosocial as well as socioeconomic stresses due to the COVID-19 pandemic in Bangladesh. Any major epidemic outbreak has negative effects on individuals and society (14), and people's fear due to COVID-19 is rational in the sense that the fatality rate of the virus is around 1% and it can kill healthy adults along with the elderly or those with existing health problems (21). It is crucial to assess the COVID-19 pandemic independently based on its attributes and not on past epidemics like SARS or MERS (22).

More than 929 COVID-19 symptom-like deaths were reported from leading newspapers and electronic media from 8th of March 2020 to 30th of April 2020. The reported case numbers certainly underestimate the actual number of infected persons given the limited number of urban testing centers, the shortage of test kits, and the long waiting times for tests and test results (9). The COVID-19 outbreak caused other critical care and infectious disease patients to be deprived of basic healthcare facilities. Patient-management decisions, early diagnosis, rapid testing, and detection are urgently needed (23, 24). The decentralization of testing and treatment facilities is required for the healthcare system to combat the pandemic. The government needs to aid in implementing testing facilities in both public and private clinical laboratories all over Bangladesh.

For a developing country, resources need to be assembled appropriately and promptly. With limited screening and testing of Covid-19 in Bangladesh, and the presence of only 48 laboratories mostly located in urban areas, it is difficult to predict when transmission of the disease will peak and when the curve will flatten (25). Predictably, community transmission in the country is happening and people are being infected and infecting their community, in some cases even without showing symptoms. It is further predicted that COVID-19 and dengue together is a deadly combination. As the monsoon season approaches, the risk of dengue infection is on the rise. It is a timely step taken by the DGHS to conduct dengue tests on suspected Covid-19 patients, as both diseases share common symptoms (reported on 9 May 2020, by DGHS in a daily press briefing on COVID-19).

### Role of Governance and Risk Communication to Reduce Societal Fear in Bangladesh

Successful governance is only possible with a competent early warning system, efficient analysis of the situation, and the interpretation, sharing, and use of relevant knowledge and information (26). Public health instructions should be established based on scientific evidence to reduce the anxiety and distress caused by misinformation and rumors. Epidemiological outcomes need to be informed on in time so that they can be accurately evaluated and explained (27). Societies where underserved communities exist strongly fear government information and politics. Public risk communications are therefore needed to prevent misinformation from social media and electronic media. The psychosocial risk (mental health impacts) for children in this situation are apparent, as they are out of touch with schools, classmates, and playmates, and deprived of physical activities and social activities; these issues need to be addressed. Moreover, the isolation and quarantine of parent/s can mentally traumatize them and result in negligence, mistreatment, and abuse in the absence of parents/caregivers (28). In addition, due to lockdown and the required maintenance of family hygiene, the burden of these activities is increased for women, considering the patriarchal nature of the country (where predominantly all household activities are performed by women). Moreover, increased levels of violence against women and girls are experienced, as in the lockdown it is almost impossible for victims to escape those family members who are the perpetrators (29). Furthermore, in the Rohingya refugee camps, it will have catastrophic outcomes (3). These kinds of risks, awareness, and prevention methods should be effectively communicated to the public.

As the pandemic continues, each new day brings in new conversations on social media and alarming developments of misinformation and propaganda, resulting in unnecessary psychological trauma and anxiety (30). Moreover, religious tension, personal tension, job insecurity, financial loss, and social insecurity could leave some people feeling particularly vulnerable and mentally unstable (22). Honest, transparent communication is vital for risk communication about the pandemic, while confusing or contradictory health messaging engenders mistrust and leads people to seek information from unreliable alternative sources and thus proliferates rumors (31).

The fear of becoming infected or fear for vulnerable family members has amplified along with the administrative procedures of testing and reluctance of other private clinics and hospitals to admit patients. At the Bbginning of this pandemic, Bangladesh had only 29 ICU beds in five dedicated hospitals in Dhaka for the treatment of COVID-19 patients. There were no ICU beds in hospitals outside Dhaka (32). This is a sign of weak governance in the healthcare system of Bangladesh. In this scenario, other critical care patients are denied admittance, experience negligence, and are often left to die without treatment. Moreover, the administrative procedure for the COVID-19 deceased, whether that be burial or cremation, has created more confusion and religious fear in the minds of the common people. Often, family members of the deceased have denied claiming the body due to fear of infection. In those cases, government authorities have intervened. Moreover, there is a rumor that the victims of COVID-19 are buried without the Muslim funeral procedures of bathing, which has created further religious tensions among people. It is, therefore, imperative that the Government manages people's fear and anxiety. Proper information should be circulated to reduce confusion. The Bangladeshi electronic and print media is not acting responsibly to disseminate truthful information and are instead reporting misguided stories on social media. Since the 26th of March, the Government of Bangladesh formed a division to monitor media to eradicate rumors or incorrect information being disseminated on social media platforms and in the mainstream media to protect the mental health of the people.

### Resilience Development in Healthcare Sectors and Probable Climatic Disaster Management

The Bangladesh Meteorological Department (BMD) had forecasted heavy rainfall events and intermittent nor'westers and cyclones at many places across the country during April and May 2020 (33). Heavy rainfall and nor'westers related to high windspeed causes tremendous disasters by destroying standing crops and properties and cause death to people and livestock.

Fair and equitable sharing of health resources could mitigate further risks to public health by meeting community health needs and generating all-important trust and resilience (31) during further climatic disasters. The development of resilience is significant to combat any disasters, even a pandemic. Subsequently, to develop resilience in the healthcare systems and to tackle any pandemic, good governance is crucial, along with good coordination. In addition, it also requires financing, service delivery, medicines and equipment for health workers, and information (34). Moreover, governments, institutions, healthcare facilities, and the general public all hold a social and ethical responsibility to assess and mitigate risks for the most vulnerable communities, including homeless people, people without adequate insurance or employment, indigenous communities, immigrant communities, people with disabilities, and certain frontline healthcare workers and emergency responders. Prisons, nursing homes, orphanages, old care homes, homeless shelters, and refugee camps can become focuses for disease outbreaks as these settings often have inadequate access to basic healthcare facilities that increases the disease burden (31). The government should prepare policies and decisions on early recovery plans which should be inclusive to all ethnic groups, religious groups, minorities, and the wide range of vulnerable populations.

April and May are the months of natural disasters like tropical cyclones, tornados, and early flooding in Bangladesh, which may be evident within the coming days. Therefore, utilization of the health-emergency disaster risk management (Health-EDRM) framework is important to implement. Health-EDRM refers to the “systematic analysis and management of health risks, posed by emergencies and disasters, through a combination of (1) hazard and vulnerability reduction to prevent and mitigate risks, (2) preparedness, (3) response and (4) recovery measures” (35). Health-EDRM is an umbrella term which the WHO uses to refer to the broad intersection of health and disaster risk management (DRM). As the patients of other seasonal diseases such as Dengue are rising, and the possibility of a natural disaster remains, the healthcare system should be coping with the changing scenario of the COVID-19 outbreak in Bangladesh, where resilience is very important. The hotspot areas of the disasters have already been identified in the Bangladesh Delta Plan 2100 (36). Vulnerable areas should be given special emphasis in the coming months for the protection of crops, risk reduction, relief preparation, and rehabilitation.

### Biomedical Waste Management Planning

Biomedical waste should be disposed of following national and international guidelines on the disposal of infectious biological hazardous materials (37). When an exponentially rapid spread of a disease or infection breaks out, the generation of biomedical waste and other related healthcare hazards may be considerably increased within a noticeably short period. If improperly treated, this waste may accelerate the spread of disease and pose a significant risk to medical staff, patients, and waste management unit personnel. A complex short-term decision-making problem is required by the authorities to deal with the fast accumulation and transportation mode of the medical waste. Healthcare centers can either directly transport the waste to the treatment centers or they can transfer and consolidate via a temporary transit center (38). The use of PPE should be distinguished by different risk factors to adopt different epidemic prevention measures and reduce the waste of personal protective equipment, as these resources are already in short supply (34). Moreover, repeated use of disposable masks and not washing cloth masks could create further risk of infection that needs to be dealt with through proper information to the public (39). As the country does not have proper incineration facilities, the government should think of setting up mobile incinerator plants rapidly to responsibly manage bio-medical waste.

### Inclusive Financing for the Disadvantaged Communities

As we have analyzed the scenario over the past months of partial, a loss of 33 billion BDT a day to GDP is incurring. More than 10 million people are becoming further marginalized due to the loss of wages and jobs (40). The dilemma of life vs. livelihoods has put people at high risk of community transmission in the industrial districts after the ready-made-garment (RMG) manufacturers trade organization BGMEA decided to open the factories even before the end of lockdown. It was predicted that the government would not get support from the allied forces. Weak governance and policy put emergency responders, such as medical doctors and healthcare staff, police, security forces, and army personnel, at risk of infection. Already, thousands of doctors and members of the police force have been infected and died during this time.

The socio-economic fall-out from this pandemic is already high, particularly for the disadvantaged poor communities, day laborers, wage earners, RMG-sector workers, and small and medium business start-ups. Already the country's RMG sector has lost many global orders due to the pandemic, and the remittance flow is at its lowest. Job insecurity and financial insecurity is foreseeable, and concerns of a global depression will affect the local market as well as investors. The prime minister of Bangladesh already declared a stimulus package of 72,750 crore BDT, of which 30,000 crore BDT has been announced for the RMG sector, other large industries, and the service sector in an attempt to defeat the economic losses due to the coronavirus situation (41). However, on priority-basis the financial incentives should be given to the poverty-stricken disadvantaged communities first, as well as insurance for healthcare professionals at the frontline, emergency responders, and caregivers responsible for emergency handling. Purchasing intensive care unit (ICU) beds, protective equipment, diagnostic test kits, mechanical ventilators, and additional supports is required for these mentally and physically affected persons who have survived COVID-19. It is also imperative to continue taking precautions, including screening, isolation of suspected cases, and social distancing, even after the pandemic is over.

Finally, combating the global pandemic is not easy. The 46 statements that we have included in this analysis aid in identifying the associations among the psychosocial, socio-economic, and possible environmental crisis based on public perception in Bangladesh. Risk mitigation measures concerning the psychosocial, socio-economic, and environmental components of the public are necessary to combat a global pandemic. Therefore, with great advancements in the speed and power of science, international collaborations are required to provide knowledge about the virus and disease recovery. Moreover, it is highly recommended by WHO and other stakeholders from the national level to raise the testing speed and facilities in Bangladesh. Multi-sectoral involvement and proper relief facilities for unprivileged populations must be ensured.

## Concluding Remarks

Without ensuring fundamental needs would be met, the lockdown due to COVID-19 has imposed mental stress on the public. The weak governance in the healthcare systems and limited healthcare facilities exacerbated the general public's fear and anxiety. The centralized COVID-19 testing facility and limitations of dedicated hospital units for COVID-19 patients hampered other critical patients from receiving healthcare services. As a country vulnerable to climate change, there might be some additional risk factors of occurring natural disasters, such as a tropical cyclone, which may add further pressure on the country. The closure of all educational institutions may increase the number of mentally depressed young people. As the business centres (except for groceries, pharmacies, and other daily necessities) are closed, it has put further stress on the country's economy. An infectious outbreak of dengue might be on the way that may have a cumulative/synergistic negative impact with COVID-19 on public health in Bangladesh. However, numerous factors that can be considered in the context of the current COVID-19 outbreak in Bangladesh are as follows: risk of community transmission, healthcare capacity, governance coordination, relief for the low-income population, biomedical waste management, and preparation for possible natural disasters. The recommendations collected in the perception study can be summarized as a need to increase COVID-testing rates and increase medical facilities. The decentralization of the COVID-19 medical facilities is particularly important due to the forced migration of more than 11 million people from Dhaka city to 64 districts of Bangladesh after the announcement of partial lockdown. In addition, proper risk assessment and dependable risk communication, a multi-sectoral management taskforce development, care of biomedical waste, ensuring basic support to vulnerable people, and good governance was suggested to reduce the psychosocial and socio-economic impact of the COVID-19 outbreak in Bangladesh. Finally, this assessment process could help the government and policymakers to judge the public perceptions to deal with the COVID-19 pandemic in densely populated lower-middle-income countries like Bangladesh.

## Data Availability Statement

All datasets presented in this study are included in the article/Supplementary Material.

## Ethics Statement

The studies involving human participants were reviewed and approved by Department of Public Health and Informatics, Jahangirnagar University, Bangladesh. The patients/participants provided their written informed consent to participate in this study.

## Author Contributions

MB-D, MS, and MR planned the studies and developed the questionnaire. Informatics and data analysis and interpretation were maintained by MB-D, AI, MS, and MR. MB and LB revised and improved the manuscript as suggested by the reviewers. All authors reviewed and read the manuscript before final submission.

## Conflict of Interest

The authors declare that the research was conducted in the absence of any commercial or financial relationships that could be construed as a potential conflict of interest.
