# Supplementary material for: Psychosocial and Socio-Economic Crisis in Bangladesh Due to COVID-19 Pandemic: A Perception-Based Assessment
Source: Front Public Health. 2020 Jun 26;8:341. doi: 10.3389/fpubh.2020.00341 (PMC7333562; doi:10.3389/fpubh.2020.00341)
Supplement: Supplementary file 1 [file Data_Sheet_1.docx]

**Table-S1: Correlation matrix of people’s perception**

|  | **MH1** | **MH2** | **MH3** | **MH4** | **MH5** | **HSB1** | **HSB2** | **HSB3** | **HSB4** | **HSB5** | **HSB6** | **HSB7** | **HSB8** | **HSB9** | **HSB10** | **GPI1** | **GPI2** | **GPI3** | **GPI4** | **GPI5** | **GPI6** | **GPI7** | **SEI1** | **SEI2** | **SEI3** | **SEI4** | **SEI5** | **SEI6** | **SEI7** | **SEI8** | **SEI9** | **SEI10** | **SEI11** | **IEI1** | **IEI2** | **IEI3** | **IEI4** | **IEI5** | **IEI6** | **IEI7** | **EEI1** | **EEI2** | **EEI3** | **EEI4** | **EEI5** | **EEI6** |
| --- | --- | --- | --- | --- | --- | --- | --- | --- | --- | --- | --- | --- | --- | --- | --- | --- | --- | --- | --- | --- | --- | --- | --- | --- | --- | --- | --- | --- | --- | --- | --- | --- | --- | --- | --- | --- | --- | --- | --- | --- | --- | --- | --- | --- | --- | --- |
| **MH1** |  |  |  |  |  |  |  |  |  |  |  |  |  |  |  |  |  |  |  |  |  |  |  |  |  |  |  |  |  |  |  |  |  |  |  |  |  |  |  |  |  |  |  |  |  |  |
| **MH2** | .584** | 1 |  |  |  |  |  |  |  |  |  |  |  |  |  |  |  |  |  |  |  |  |  |  |  |  |  |  |  |  |  |  |  |  |  |  |  |  |  |  |  |  |  |  |  |  |
| **MH3** | .511** | .654** | 1 |  |  |  |  |  |  |  |  |  |  |  |  |  |  |  |  |  |  |  |  |  |  |  |  |  |  |  |  |  |  |  |  |  |  |  |  |  |  |  |  |  |  |  |
| **MH4** | .429** | .391** | .341** | 1 |  |  |  |  |  |  |  |  |  |  |  |  |  |  |  |  |  |  |  |  |  |  |  |  |  |  |  |  |  |  |  |  |  |  |  |  |  |  |  |  |  |  |
| **MH5** | .238** | .243** | .211** | .344** | 1 |  |  |  |  |  |  |  |  |  |  |  |  |  |  |  |  |  |  |  |  |  |  |  |  |  |  |  |  |  |  |  |  |  |  |  |  |  |  |  |  |  |
| **HSB1** | .218** | .132** | .161** | .191** | .155** | 1 |  |  |  |  |  |  |  |  |  |  |  |  |  |  |  |  |  |  |  |  |  |  |  |  |  |  |  |  |  |  |  |  |  |  |  |  |  |  |  |  |
| **HSB2** | .182** | .128** | .132** | .222** | .155** | .277** | 1 |  |  |  |  |  |  |  |  |  |  |  |  |  |  |  |  |  |  |  |  |  |  |  |  |  |  |  |  |  |  |  |  |  |  |  |  |  |  |  |
| **HSB3** | .202** | .165** | .166** | .220** | .189** | .255** | .326** | 1 |  |  |  |  |  |  |  |  |  |  |  |  |  |  |  |  |  |  |  |  |  |  |  |  |  |  |  |  |  |  |  |  |  |  |  |  |  |  |
| **HSB4** | .132** | 0.058 | .071* | .093** | .095** | .205** | .227** | .295** | 1 |  |  |  |  |  |  |  |  |  |  |  |  |  |  |  |  |  |  |  |  |  |  |  |  |  |  |  |  |  |  |  |  |  |  |  |  |  |
| **HSB5** | .214** | .170** | .171** | .182** | .186** | .388** | .320** | .392** | .395** | 1 |  |  |  |  |  |  |  |  |  |  |  |  |  |  |  |  |  |  |  |  |  |  |  |  |  |  |  |  |  |  |  |  |  |  |  |  |
| **HSB6** | .206** | .164** | .176** | .175** | .192** | .305** | .294** | .356** | .395** | .644** | 1 |  |  |  |  |  |  |  |  |  |  |  |  |  |  |  |  |  |  |  |  |  |  |  |  |  |  |  |  |  |  |  |  |  |  |  |
| **HSB7** | .198** | .118** | .181** | .152** | .184** | .337** | .323** | .382** | .363** | .585** | .551** | 1 |  |  |  |  |  |  |  |  |  |  |  |  |  |  |  |  |  |  |  |  |  |  |  |  |  |  |  |  |  |  |  |  |  |  |
| **HSB8** | .197** | .130** | .149** | .124** | .150** | .274** | .278** | .348** | .326** | .598** | .536** | .565** | 1 |  |  |  |  |  |  |  |  |  |  |  |  |  |  |  |  |  |  |  |  |  |  |  |  |  |  |  |  |  |  |  |  |  |
| **HSB9** | .104** | .120** | .107** | .137** | .110** | .116** | .188** | .239** | .261** | .277** | .325** | .274** | .279** | 1 |  |  |  |  |  |  |  |  |  |  |  |  |  |  |  |  |  |  |  |  |  |  |  |  |  |  |  |  |  |  |  |  |
| **HSB10** | .178** | .175** | .194** | .188** | .211** | .253** | .250** | .345** | .257** | .458** | .454** | .435** | .468** | .286** | 1 |  |  |  |  |  |  |  |  |  |  |  |  |  |  |  |  |  |  |  |  |  |  |  |  |  |  |  |  |  |  |  |
| **GPI1** | -.071* | -0.054 | -0.052 | -0.043 | 0.017 | -.085** | -.087** | -.092** | -.114** | -.100** | -.104** | -.105** | -.063* | -.199** | -.091** | 1 |  |  |  |  |  |  |  |  |  |  |  |  |  |  |  |  |  |  |  |  |  |  |  |  |  |  |  |  |  |  |
| **GPI2** | -.112** | -.094** | -.140** | -0.002 | .064* | -.128** | 0.021 | -.113** | -0.007 | -.107** | -.084** | -.093** | -.065* | -0.022 | -.112** | .367** | 1 |  |  |  |  |  |  |  |  |  |  |  |  |  |  |  |  |  |  |  |  |  |  |  |  |  |  |  |  |  |
| **GPI3** | -.114** | -.096** | -.143** | -0.038 | 0.021 | -.185** | -0.055 | -.125** | -.069* | -.138** | -.133** | -.126** | -.140** | -.063* | -.136** | .344** | .605** | 1 |  |  |  |  |  |  |  |  |  |  |  |  |  |  |  |  |  |  |  |  |  |  |  |  |  |  |  |  |
| **GPI4** | -0.056 | -0.042 | -0.046 | 0.005 | .067* | -.083** | 0.045 | -.086** | -0.051 | -0.025 | -0.053 | -0.049 | 0.017 | -0.005 | -.071* | .242** | .497** | .471** | 1 |  |  |  |  |  |  |  |  |  |  |  |  |  |  |  |  |  |  |  |  |  |  |  |  |  |  |  |
| **GPI5** | .138** | .076* | .118** | .150** | .167** | .171** | .271** | .224** | .166** | .234** | .264** | .270** | .312** | .061* | .331** | 0.047 | .189** | .066* | .164** | 1 |  |  |  |  |  |  |  |  |  |  |  |  |  |  |  |  |  |  |  |  |  |  |  |  |  |  |
| **GPI6** | .177** | .115** | .156** | .166** | .173** | .225** | .234** | .257** | .133** | .338** | .305** | .321** | .375** | .121** | .434** | 0.016 | 0.019 | -.098** | 0.026 | .541** | 1 |  |  |  |  |  |  |  |  |  |  |  |  |  |  |  |  |  |  |  |  |  |  |  |  |  |
| **GPI7** | .146** | .123** | .117** | .099** | .126** | 0.02 | .074* | .079** | .092** | .094** | .088** | .070* | .086** | .078* | .145** | .159** | .168** | .152** | .144** | .146** | .181** | 1 |  |  |  |  |  |  |  |  |  |  |  |  |  |  |  |  |  |  |  |  |  |  |  |  |
| **SEI1** | .165** | .178** | .163** | .110** | .080** | .144** | .238** | .189** | .068* | .237** | .146** | .210** | .194** | 0.015 | .210** | 0.057 | .077* | 0.037 | 0.054 | .296** | .295** | .140** | 1 |  |  |  |  |  |  |  |  |  |  |  |  |  |  |  |  |  |  |  |  |  |  |  |
| **SEI2** | .109** | .075* | .113** | .110** | .183** | .162** | .216** | .194** | .101** | .241** | .184** | .231** | .220** | .106** | .240** | .063* | .084** | 0.037 | .063* | .287** | .263** | .084** | .231** | 1 |  |  |  |  |  |  |  |  |  |  |  |  |  |  |  |  |  |  |  |  |  |  |
| **SEI3** | .104** | .078* | .080** | .161** | .218** | .174** | .241** | .235** | .100** | .242** | .240** | .293** | .239** | .125** | .250** | -.065* | 0.03 | -0.048 | 0.001 | .319** | .303** | 0.059 | .243** | .636** | 1 |  |  |  |  |  |  |  |  |  |  |  |  |  |  |  |  |  |  |  |  |  |
| **SEI4** | .150** | .179** | .105** | .151** | .185** | .168** | .222** | .283** | .121** | .266** | .209** | .254** | .264** | .158** | .334** | -0.037 | -0.006 | -.096** | -0.005 | .310** | .362** | .100** | .261** | .419** | .487** | 1 |  |  |  |  |  |  |  |  |  |  |  |  |  |  |  |  |  |  |  |  |
| **SEI5** | .069* | .070* | .097** | .127** | .151** | .104** | .154** | .234** | .131** | .141** | .136** | .213** | .224** | .150** | .265** | 0.005 | 0.012 | -0.019 | 0.018 | .146** | .221** | .118** | .163** | .284** | .345** | .405** | 1 |  |  |  |  |  |  |  |  |  |  |  |  |  |  |  |  |  |  |  |
| **SEI6** | .113** | .131** | .149** | .118** | .133** | .150** | .186** | .201** | .081** | .182** | .190** | .219** | .198** | .158** | .289** | -0.053 | -.084** | -.088** | -0.04 | .131** | .220** | .122** | .174** | .344** | .412** | .432** | .479** | 1 |  |  |  |  |  |  |  |  |  |  |  |  |  |  |  |  |  |  |
| **SEI7** | 0.048 | .093** | .127** | .132** | .105** | .113** | .155** | .217** | .120** | .164** | .154** | .143** | .141** | .214** | .248** | -0.041 | -0.045 | -.069* | -0.034 | .072* | .150** | .077* | 0.049 | .247** | .249** | .270** | .327** | .424** | 1 |  |  |  |  |  |  |  |  |  |  |  |  |  |  |  |  |  |
| **SEI8** | 0.058 | .083** | .079* | .143** | .094** | .146** | .185** | .196** | .101** | .203** | .163** | .204** | .219** | .150** | .242** | -.096** | -.086** | -.141** | -0.056 | .114** | .210** | -0.002 | .121** | .288** | .298** | .320** | .294** | .363** | .504** | 1 |  |  |  |  |  |  |  |  |  |  |  |  |  |  |  |  |
| **SEI9** | .082** | .107** | .127** | .128** | .180** | .138** | .174** | .250** | .109** | .274** | .217** | .279** | .301** | .175** | .362** | -.061* | -0.049 | -.103** | -0.034 | .278** | .342** | .106** | .225** | .369** | .420** | .540** | .375** | .459** | .420** | .440** | 1 |  |  |  |  |  |  |  |  |  |  |  |  |  |  |  |
| **SEI10** | 0.046 | .084** | .084** | .106** | .182** | .112** | .154** | .152** | .127** | .171** | .156** | .217** | .162** | .146** | .191** | -0.021 | .078* | 0.01 | 0.05 | .202** | .220** | .129** | .176** | .355** | .399** | .331** | .289** | .300** | .287** | .268** | .402** | 1 |  |  |  |  |  |  |  |  |  |  |  |  |  |  |
| **SEI11** | .142** | .116** | .154** | .157** | .155** | .160** | .130** | .151** | .109** | .164** | .194** | .185** | .158** | .213** | .225** | -0.013 | -.084** | -0.056 | -0.045 | .066* | .138** | .113** | 0.034 | .267** | .274** | .221** | .291** | .373** | .364** | .316** | .298** | .367** | 1 |  |  |  |  |  |  |  |  |  |  |  |  |  |
| **IEI1** | .315** | .240** | .198** | .180** | .106** | .268** | .192** | .228** | .136** | .266** | .267** | .327** | .240** | .111** | .287** | -.079* | -.116** | -.149** | -.071* | .227** | .244** | .108** | .186** | .227** | .224** | .234** | .129** | .264** | .139** | .145** | .204** | .175** | .233** | 1 |  |  |  |  |  |  |  |  |  |  |  |  |
| **IEI2** | .366** | .319** | .330** | .219** | .098** | .186** | .180** | .281** | .109** | .272** | .292** | .216** | .232** | .154** | .300** | -.118** | -.194** | -.177** | -.077* | .191** | .257** | .162** | .159** | .163** | .193** | .234** | .153** | .236** | .182** | .169** | .210** | .178** | .182** | .492** | 1 |  |  |  |  |  |  |  |  |  |  |  |
| **IEI3** | .263** | .215** | .208** | .183** | .127** | .286** | .237** | .321** | .196** | .400** | .342** | .381** | .354** | .122** | .386** | -.147** | -.160** | -.263** | -.132** | .282** | .369** | 0.041 | .182** | .255** | .279** | .316** | .195** | .270** | .180** | .238** | .310** | .205** | .145** | .457** | .446** | 1 |  |  |  |  |  |  |  |  |  |  |
| **IEI4** | .301** | .256** | .267** | .196** | .185** | .295** | .240** | .292** | .198** | .428** | .366** | .368** | .417** | .153** | .443** | -.114** | -.150** | -.185** | -.071* | .338** | .425** | .125** | .237** | .248** | .288** | .318** | .207** | .295** | .198** | .217** | .320** | .253** | .181** | .446** | .524** | .597** | 1 |  |  |  |  |  |  |  |  |  |
| **IEI5** | .266** | .262** | .258** | .257** | .209** | .261** | .257** | .303** | .200** | .346** | .346** | .405** | .345** | .175** | .386** | -.076* | -.108** | -.121** | -.068* | .310** | .370** | .109** | .206** | .243** | .291** | .302** | .262** | .248** | .178** | .204** | .298** | .280** | .200** | .388** | .424** | .502** | .594** | 1 |  |  |  |  |  |  |  |  |
| **IEI6** | .202** | .177** | .164** | .229** | .181** | .170** | .238** | .220** | .093** | .260** | .225** | .263** | .240** | .101** | .276** | -0.021 | -0.024 | -0.039 | 0.016 | .247** | .305** | .183** | .213** | .205** | .250** | .271** | .236** | .268** | .246** | .246** | .308** | .285** | .240** | .299** | .281** | .312** | .385** | .430** | 1 |  |  |  |  |  |  |  |
| **IEI7** | .158** | .135** | .178** | .173** | .101** | .205** | .113** | .190** | .096** | .220** | .227** | .217** | .212** | .169** | .250** | -.113** | -.309** | -.274** | -.183** | 0.036 | .204** | .062* | 0.051 | .156** | .163** | .159** | .169** | .249** | .223** | .247** | .196** | .130** | .253** | .263** | .325** | .300** | .315** | .334** | .310** | 1 |  |  |  |  |  |  |
| **EEI1** | .149** | .172** | .185** | .152** | .102** | .135** | .174** | .153** | .118** | .108** | .140** | .165** | .109** | .132** | .161** | -0.045 | -.066* | -0.033 | -0.032 | 0.048 | .081** | .148** | .082** | .140** | .141** | .117** | .138** | .219** | .192** | .128** | .162** | .192** | .224** | .190** | .174** | .109** | .166** | .203** | .239** | .245** | 1 |  |  |  |  |  |
| **EEI2** | .194** | .141** | .184** | .161** | .161** | .219** | .283** | .257** | .201** | .327** | .315** | .323** | .275** | .155** | .303** | -.090** | -.098** | -.131** | -0.034 | .270** | .322** | .118** | .176** | .315** | .343** | .363** | .238** | .276** | .229** | .241** | .385** | .306** | .210** | .303** | .289** | .381** | .421** | .422** | .321** | .255** | .301** | 1 |  |  |  |  |
| **EEI3** | .141** | .146** | .172** | .153** | .111** | .161** | .227** | .222** | .130** | .203** | .248** | .246** | .197** | .208** | .264** | -.072* | -.068* | -0.056 | 0.011 | .122** | .155** | .099** | .087** | .204** | .235** | .211** | .229** | .256** | .351** | .286** | .288** | .261** | .252** | .202** | .230** | .228** | .284** | .288** | .264** | .181** | .310** | .417** | 1 |  |  |  |
| **EEI4** | .149** | .164** | .178** | .179** | .180** | .236** | .250** | .239** | .169** | .268** | .271** | .280** | .234** | .202** | .331** | -.087** | -.063* | -.088** | -0.038 | .231** | .222** | .099** | .164** | .380** | .447** | .379** | .319** | .350** | .274** | .269** | .349** | .344** | .249** | .252** | .245** | .291** | .367** | .356** | .279** | .194** | .229** | .481** | .485** | 1 |  |  |
| **EEI5** | .136** | .167** | .186** | .186** | .200** | .225** | .211** | .226** | .127** | .264** | .276** | .269** | .234** | .184** | .340** | -.081** | -.082** | -.075* | -0.032 | .189** | .266** | .132** | .177** | .344** | .406** | .386** | .345** | .421** | .330** | .314** | .411** | .339** | .297** | .259** | .303** | .298** | .394** | .358** | .289** | .275** | .266** | .425** | .464** | .667** | 1 |  |
| **EEI6** | .157** | .162** | .162** | .165** | .146** | .244** | .270** | .257** | .193** | .317** | .291** | .336** | .297** | .181** | .336** | -.113** | -.075* | -.124** | -.064* | .245** | .295** | .121** | .207** | .389** | .421** | .363** | .279** | .387** | .332** | .353** | .404** | .366** | .313** | .330** | .316** | .387** | .445** | .405** | .332** | .299** | .244** | .473** | .471** | .630** | .659** | 1 |

** Correlation is significant at the 0.01 level (2-tailed). * Correlation is significant at the 0.05 level (2-tailed).

**Table-S2: Descriptive overview of the items**

| **Items** | **Mean** | **Std. Error of Mean** | **Median** | **Mode** | **Std. Deviation** | **Skewness** | **Std. Error of Skewness** | **Kurtosis** | **Std. Error of Kurtosis** |
| --- | --- | --- | --- | --- | --- | --- | --- | --- | --- |
| MH1 | 4.152 | 0.03101 | 4 | 5 | 1.01231 | -1.271 | 0.075 | 1.203 | 0.15 |
| MH2 | 3.8386 | 0.03308 | 4 | 4 | 1.08001 | -0.747 | 0.075 | -0.07 | 0.15 |
| MH3 | 4.0854 | 0.0333 | 4 | 5 | 1.08737 | -1.131 | 0.075 | 0.525 | 0.15 |
| MH4 | 4.0713 | 0.03219 | 4 | 5 | 1.05108 | -1.11 | 0.075 | 0.659 | 0.15 |
| MH5 | 4.1951 | 0.03223 | 5 | 5 | 1.05235 | -1.369 | 0.075 | 1.301 | 0.15 |
| HSB1 | 4.3593 | 0.03092 | 5 | 5 | 1.00953 | -1.736 | 0.075 | 2.476 | 0.15 |
| HSB2 | 4.515 | 0.02675 | 5 | 5 | 0.87332 | -2.237 | 0.075 | 5.195 | 0.15 |
| HSB3 | 4.5488 | 0.02378 | 5 | 5 | 0.77639 | -2.147 | 0.075 | 5.378 | 0.15 |
| HSB4 | 4.227 | 0.03089 | 5 | 5 | 1.00844 | -1.374 | 0.075 | 1.451 | 0.15 |
| HSB5 | 4.6482 | 0.02247 | 5 | 5 | 0.73356 | -2.645 | 0.075 | 8.058 | 0.15 |
| HSB6 | 4.606 | 0.02375 | 5 | 5 | 0.77552 | -2.432 | 0.075 | 6.482 | 0.15 |
| HSB7 | 4.5694 | 0.02247 | 5 | 5 | 0.73374 | -1.927 | 0.075 | 4.103 | 0.15 |
| HSB8 | 4.7223 | 0.02189 | 5 | 5 | 0.71458 | -3.295 | 0.075 | 12.001 | 0.15 |
| HSB9 | 4.0544 | 0.03631 | 4 | 5 | 1.18553 | -1.162 | 0.075 | 0.354 | 0.15 |
| HSB10 | 4.6426 | 0.0227 | 5 | 5 | 0.74103 | -2.495 | 0.075 | 6.901 | 0.15 |
| GPI1 | 2.5056 | 0.03931 | 2 | 1 | 1.28351 | 0.436 | 0.075 | -0.905 | 0.15 |
| GPI2 | 2.7617 | 0.03851 | 3 | 2 | 1.25727 | 0.121 | 0.075 | -1.088 | 0.15 |
| GPI3 | 2.272 | 0.03646 | 2 | 1 | 1.19038 | 0.664 | 0.075 | -0.52 | 0.15 |
| GPI4 | 2.9794 | 0.03455 | 3 | 3 | 1.12819 | -0.105 | 0.075 | -0.654 | 0.15 |
| GPI5 | 4.5657 | 0.02378 | 5 | 5 | 0.77635 | -2.319 | 0.075 | 6.367 | 0.15 |
| GPI6 | 4.6754 | 0.02115 | 5 | 5 | 0.69044 | -2.73 | 0.075 | 8.888 | 0.15 |
| GPI7 | 3.4512 | 0.03084 | 3 | 3 | 1.007 | -0.308 | 0.075 | -0.156 | 0.15 |
| SEI1 | 4.5338 | 0.02369 | 5 | 5 | 0.77356 | -2.186 | 0.075 | 5.877 | 0.15 |
| SEI2 | 4.5113 | 0.02371 | 5 | 5 | 0.77421 | -1.935 | 0.075 | 4.453 | 0.15 |
| SEI3 | 4.5084 | 0.02202 | 5 | 5 | 0.71891 | -1.701 | 0.075 | 3.719 | 0.15 |
| SEI4 | 4.788 | 0.01849 | 5 | 5 | 0.60366 | -3.639 | 0.075 | 15.449 | 0.15 |
| SEI5 | 4.4203 | 0.02681 | 5 | 5 | 0.87518 | -1.7 | 0.075 | 2.797 | 0.15 |
| SEI6 | 4.3621 | 0.02622 | 5 | 5 | 0.85593 | -1.515 | 0.075 | 2.416 | 0.15 |
| SEI7 | 4.1295 | 0.02974 | 4 | 5 | 0.971 | -1.149 | 0.075 | 0.956 | 0.15 |
| SEI8 | 4.2889 | 0.02715 | 5 | 5 | 0.88652 | -1.303 | 0.075 | 1.46 | 0.15 |
| SEI9 | 4.6013 | 0.0218 | 5 | 5 | 0.71178 | -2.301 | 0.075 | 6.604 | 0.15 |
| SEI10 | 4.379 | 0.02686 | 5 | 5 | 0.87688 | -1.628 | 0.075 | 2.756 | 0.15 |
| SEI11 | 3.9024 | 0.0327 | 4 | 5 | 1.0676 | -0.863 | 0.075 | 0.201 | 0.15 |
| IEI1 | 4.3893 | 0.02503 | 5 | 5 | 0.81732 | -1.373 | 0.075 | 1.897 | 0.15 |
| IEI2 | 4.2083 | 0.02837 | 4 | 5 | 0.92628 | -1.056 | 0.075 | 0.639 | 0.15 |
| IEI3 | 4.5572 | 0.02354 | 5 | 5 | 0.76852 | -1.99 | 0.075 | 4.303 | 0.15 |
| IEI4 | 4.5629 | 0.02253 | 5 | 5 | 0.73561 | -2.04 | 0.075 | 4.96 | 0.15 |
| IEI5 | 4.5066 | 0.0223 | 5 | 5 | 0.72801 | -1.698 | 0.075 | 3.573 | 0.15 |
| IEI6 | 4.2842 | 0.02449 | 4 | 5 | 0.79957 | -1.064 | 0.075 | 1.111 | 0.15 |
| IEI7 | 3.8293 | 0.03424 | 4 | 5 | 1.1179 | -0.642 | 0.075 | -0.362 | 0.15 |
| EEI1 | 3.6979 | 0.03074 | 4 | 3 | 1.00363 | -0.417 | 0.075 | -0.254 | 0.15 |
| EEI2 | 4.6501 | 0.0207 | 5 | 5 | 0.67586 | -2.202 | 0.075 | 5.301 | 0.15 |
| EEI3 | 4.3002 | 0.02574 | 5 | 5 | 0.84027 | -1.116 | 0.075 | 0.89 | 0.15 |
| EEI4 | 4.5901 | 0.02026 | 5 | 5 | 0.66138 | -1.875 | 0.075 | 4.59 | 0.15 |
| EEI5 | 4.4306 | 0.02399 | 5 | 5 | 0.78325 | -1.449 | 0.075 | 2.065 | 0.15 |
| EEI6 | 4.485 | 0.02214 | 5 | 5 | 0.72271 | -1.57 | 0.075 | 3.004 | 0.15 |

**Table-S3: One sample T-test**

| **Items** | **t** | **df** | **Sig. (2-tailed)** | **Mean Difference** | **95% Confidence Interval of the Difference** | |
| --- | --- | --- | --- | --- | --- | --- |
|  |  |  |  |  | **Lower** | **Upper** |
| MH1 | 133.912 | 1065 | 0 | 4.15197 | 4.0911 | 4.2128 |
| MH2 | 116.045 | 1065 | 0 | 3.83865 | 3.7737 | 3.9036 |
| MH3 | 122.668 | 1065 | 0 | 4.08537 | 4.02 | 4.1507 |
| MH4 | 126.466 | 1065 | 0 | 4.07129 | 4.0081 | 4.1345 |
| MH5 | 130.156 | 1065 | 0 | 4.19512 | 4.1319 | 4.2584 |
| HSB1 | 140.986 | 1065 | 0 | 4.35929 | 4.2986 | 4.42 |
| HSB2 | 168.797 | 1065 | 0 | 4.51501 | 4.4625 | 4.5675 |
| HSB3 | 191.29 | 1065 | 0 | 4.54878 | 4.5021 | 4.5954 |
| HSB4 | 136.855 | 1065 | 0 | 4.22702 | 4.1664 | 4.2876 |
| HSB5 | 206.886 | 1065 | 0 | 4.64822 | 4.6041 | 4.6923 |
| HSB6 | 193.915 | 1065 | 0 | 4.606 | 4.5594 | 4.6526 |
| HSB7 | 203.329 | 1065 | 0 | 4.56942 | 4.5253 | 4.6135 |
| HSB8 | 215.768 | 1065 | 0 | 4.72233 | 4.6794 | 4.7653 |
| HSB9 | 111.659 | 1065 | 0 | 4.05441 | 3.9832 | 4.1257 |
| HSB10 | 204.55 | 1065 | 0 | 4.64259 | 4.5981 | 4.6871 |
| GPI1 | 63.738 | 1065 | 0 | 2.50563 | 2.4285 | 2.5828 |
| GPI2 | 71.718 | 1065 | 0 | 2.76173 | 2.6862 | 2.8373 |
| GPI3 | 62.318 | 1065 | 0 | 2.27205 | 2.2005 | 2.3436 |
| GPI4 | 86.222 | 1065 | 0 | 2.97936 | 2.9116 | 3.0472 |
| GPI5 | 192.01 | 1065 | 0 | 4.56567 | 4.519 | 4.6123 |
| GPI6 | 221.092 | 1065 | 0 | 4.67542 | 4.6339 | 4.7169 |
| GPI7 | 111.898 | 1065 | 0 | 3.45122 | 3.3907 | 3.5117 |
| SEI1 | 191.358 | 1065 | 0 | 4.53377 | 4.4873 | 4.5803 |
| SEI2 | 190.246 | 1065 | 0 | 4.51126 | 4.4647 | 4.5578 |
| SEI3 | 204.753 | 1065 | 0 | 4.50844 | 4.4652 | 4.5516 |
| SEI4 | 258.966 | 1065 | 0 | 4.78799 | 4.7517 | 4.8243 |
| SEI5 | 164.904 | 1065 | 0 | 4.42026 | 4.3677 | 4.4729 |
| SEI6 | 166.394 | 1065 | 0 | 4.3621 | 4.3107 | 4.4135 |
| SEI7 | 138.851 | 1065 | 0 | 4.12946 | 4.0711 | 4.1878 |
| SEI8 | 157.956 | 1065 | 0 | 4.28893 | 4.2357 | 4.3422 |
| SEI9 | 211.065 | 1065 | 0 | 4.60131 | 4.5585 | 4.6441 |
| SEI10 | 163.048 | 1065 | 0 | 4.37899 | 4.3263 | 4.4317 |
| SEI11 | 119.346 | 1065 | 0 | 3.90244 | 3.8383 | 3.9666 |
| IEI1 | 175.34 | 1065 | 0 | 4.38931 | 4.3402 | 4.4384 |
| IEI2 | 148.333 | 1065 | 0 | 4.20826 | 4.1526 | 4.2639 |
| IEI3 | 193.609 | 1065 | 0 | 4.55722 | 4.511 | 4.6034 |
| IEI4 | 202.521 | 1065 | 0 | 4.56285 | 4.5186 | 4.6071 |
| IEI5 | 202.109 | 1065 | 0 | 4.50657 | 4.4628 | 4.5503 |
| IEI6 | 174.942 | 1065 | 0 | 4.28424 | 4.2362 | 4.3323 |
| IEI7 | 111.839 | 1065 | 0 | 3.82927 | 3.7621 | 3.8965 |
| EEI1 | 120.3 | 1065 | 0 | 3.69794 | 3.6376 | 3.7583 |
| EEI2 | 224.637 | 1065 | 0 | 4.65009 | 4.6095 | 4.6907 |
| EEI3 | 167.09 | 1065 | 0 | 4.30019 | 4.2497 | 4.3507 |
| EEI4 | 226.593 | 1065 | 0 | 4.59006 | 4.5503 | 4.6298 |
| EEI5 | 184.687 | 1065 | 0 | 4.43058 | 4.3835 | 4.4777 |
| EEI6 | 202.617 | 1065 | 0 | 4.48499 | 4.4416 | 4.5284 |
